# Tribbles Homolog 3 Involved in Radiation Response of Triple Negative Breast Cancer Cells by Regulating Notch1 Activation

**DOI:** 10.3390/cancers11020127

**Published:** 2019-01-22

**Authors:** Yueh-Chun Lee, Wen-Ling Wang, Wei-Chao Chang, Yu-Hao Huang, Guan-Ci Hong, Hui-Lin Wang, Ying-Hsiang Chou, Hsien-Chun Tseng, Hsueh-Te Lee, Shao-Ti Li, Hsin-Lin Chen, Chun-Chieh Wu, Huei-Fan Yang, Bing-Yen Wang, Wen-Wei Chang

**Affiliations:** 1 Department of Radiation Oncology, Chung Shan Medical University Hospital, Taichung 40201, Taiwan; lee.yuehchun@gmail.com (Y.-C.L.); hideka.chou@gmail.com (Y.-H.C.); rad.tseng@msa.hinet.net (H.-C.T.); showtear@gmail.com (S.-T.L.); cznpzz@gmail.com (H.-L.C.); kubernet332@gmail.com (C.-C.W.); benlinda.tw@yahoo.com.tw (H.-F.Y.); 2 School of Biomedical Sciences, Chung Shan Medical University, Taichung 40201, Taiwan; u8966003.lin@gmail.com (W.-L.W.); bms0217062@gmail.com (Y.-H.H.); cream8515@gmail.com (G.-C.H.); huilin117@gmail.com (H.-L.W.); 3 Graduate Institute of Biomedical Sciences and Center for Molecular Medicine, China Medical University, Taichung 40402, Taiwan; proma@mail.cmu.edu.tw; 4 Department of Medical Imaging and Radiological Sciences, Chung Shan Medical University, Taichung 40201, Taiwan; 5 School of Medicine, Chung Shan Medical University, Taichung 40201, Taiwan; 6 Institute of Anatomy and Cell Biology, School of Medicine, National Yang Ming University, Taipei 11221, Taiwan; incubator.lee@ym.edu.tw; 7 Department of Nursing, Chung shan Medical University Hospital, Taichung 40201, Taiwan; 8 Division of Thoracic Surgery, Department of Surgery, Changhua Christian Hospital, Changhua City 50006, Taiwan; 9 Center for General Education, Ming Dao University, Changhua 52345, Taiwan; 10 School of Medicine, Chung Shan Medical University, Taichung 40201, Taiwan; 11 Institute of Genomics and Bioinformatics, National Chung Hsing University, Taichung 40201, Taiwan; 12 School of Medicine, College of Medicine, Kaohsiung Medical University, Kaohsiung 80708, Taiwan; 13 Ph.D. Program in Translational Medicine, National Chung Hsing University, Taichung 40201, Taiwan; 14 Department of Medical Research, Chung Shan Medical University Hospital, Taichung 40201, Taiwan

**Keywords:** TRIB3, triple negative breast cancer, radioresistance, Notch1

## Abstract

Breast cancer is the most common cancer for women in Taiwan and post-lumpectomy radiotherapy is one of the therapeutic strategies for this malignancy. Although the 10-year overall survival of breast cancer patients is greatly improved by radiotherapy, the locoregional recurrence is around 10% and triple negative breast cancers (TNBCs) are at a high risk for relapse. The aim of this paper is to understand the mechanisms of radioresistance in breast cancers which may facilitate the development of new treatments in sensitizing breast cancer toward radiation therapy. Tribbles homolog 3 (TRIB3) is a pseudokinase protein and known to function as a protein scaffold within cells. It has been reported that higher TRIB3 expression is a poor prognostic factor in breast cancer patients with radiotherapy. In this study, we investigate the involvement of TRIB3 in the radiation response of TNBC cells. We first found that the expression of TRIB3 and the activation of Notch1, as well as Notch1 target genes, increased in two radioresistant TNBC cells. Knockdown of TRIB3 in radioresistant MDA-MB-231 TNBC cells decreased Notch1 activation, as well as the CD24-CD44^+^ cancer stem cell population, and sensitized cells toward radiation treatment. The inhibitory effects of TRIB3 knockdown in self-renewal or radioresistance could be reversed by forced expression of the Notch intracellular domain. We also observed an inhibition in cell growth and accumulated cells in the G_0_/G^1^ phase in radioresistant MDA-MB-231 cells after knockdown of TRIB3. With immunoprecipitation and mass spectrometry analysis, we found that, BCL2-associated transcription factor 1 (BCLAF1), BCL2 interacting protein 1 (BNIP1), or DEAD-box helicase 5 (DDX5) were the possible TRIB3 interacting proteins and immunoprecipitation data also confirmed that these proteins interacted with TRIB3 in radioresistant MDA-MB-231 cells. In conclusion, the expression of TRIB3 in radioresistant TNBC cells participated in Notch1 activation and targeted TRIB3 expression may be a strategy to sensitize TNBC cells toward radiation therapy.

## 1. Introduction

Breast cancer is the most common cancer for women in Taiwan. Surgery remains the most important primary treatment for breast cancer, which is to remove most of the cancer cells from the breast. Systemic treatment, including chemotherapy, hormone therapy, and target therapy, also plays an important role in the treatment of breast cancer to decrease both the local and distant recurrence rate [1]. Radiotherapy was added for breast cancer patients who have higher local recurrence rate after the surgery in order to further improve the local control and overall survival. The Early Breast Cancer Trialists’ Collaborative Group (EBCTCG), who conducted a meta-analysis to evaluate the effect of radiotherapy for women receiving breast conserving surgery, reported that adding radiotherapy to patients after the breast conserving surgery can reduce the 10-year risk of locoregional recurrence rate from 25% to 8% [2]. Despite the use of radiotherapy in breast cancer treatment, the locoregional recurrence rate in 10 years was still among 4–13% for different patient populations [3]. The factors affecting the recurrence rate for breast cancer includes young age at diagnosis (<40), negative for estrogen receptor (ER) status, negative for progesterone receptor (PR), positive for human epidermal growth factor receptor 2 (HER2), and triple negative for ER, PR, and HER2 receptors, as well as locally advanced stage (tumor <5 cm or positive lymph node involvement) [4]. Among the subtypes of breast cancer, triple negative breast cancers (TNBCs), which express very low levels or are negative for ER, PR, and HER2, are significantly associated with a high risk of locoregional recurrence following radiotherapy [5]. Thus, TNBCs are considered to be more insensitive to radiotherapy than other subtypes of breast cancer [5] and the molecular mechanisms in TNBC cells resistant to radiotherapy are not completely understood. 

Tribbles homolog 3 (TRIB3) is a pseudokinase protein without kinase activity. The role of TRIB3 in cancer development is controversial. It has been reported that TRIB3 could directly bind to RAC-α serine/threonine-protein kinase (AKT1) and suppress its kinase activity [6]. TRIB3 also acts as a negative regulator of nuclear factor κ-light-chain-enhancer of activated B cells (NF-κB) [7]. However, TRIB3 has been reported to be overexpressed in several cancer types, such as breast cancer [8] and colorectal cancer [9]. TRIB3 could interact with SMAD family member 3 (SMAD3) to promote transforming growth factor (TGF)-β induced tumor cell migration and invasion. [10]. TRIB3 has also been demonstrated to interact with p62 and lead to the inhibition of autophagic degradation of several oncogenic proteins. Disruption of the interaction between TRIB3 and p62 resulted in a tumor suppression consequence in an animal model [11]. In breast cancer, Wennemers et al. [12] reported that TRIB3 was a poor prognostic factor within patients receiving radiotherapy and could be induced by hypoxia. However, the direct involvement of TRIB3 in radioresistant breast cancer cells remains unclear.

In this present study, we first established two radioresistant TNBC cells by repeated exposure of 2 Gy radiation. With quantitative real time (RT)-PCR and Western blot analysis, we found the expression of TRIB3 and the activation of *NOTCH1* was increased in radioresistant TNBC cells. Applying RNA interference to knockdown TRIB3 expression resulted in the downregulation of Notch1 activation and sensitized radioresistant MDA-MB-231 TNBC cells toward radiation treatment. We also discovered by mass spectrometry and Western blot analysis that BCL2-associated transcription factor 1 (BCLAF1), BCL2 interacting protein 1 (BNIP1), or DEAD-box helicase 5 (DDX5) might be the TRIB3 interacting proteins. Our data suggest that targeting TRIB3 in TNBC cells may be a strategy in sensitizing these cells toward radiation therapy.

## 2. Results

### 2.1. TRIB3 and Notch1 Activation is Upregulated in Radioresistant Triple Negative Breast Cancer Cells

**Figure 1:**
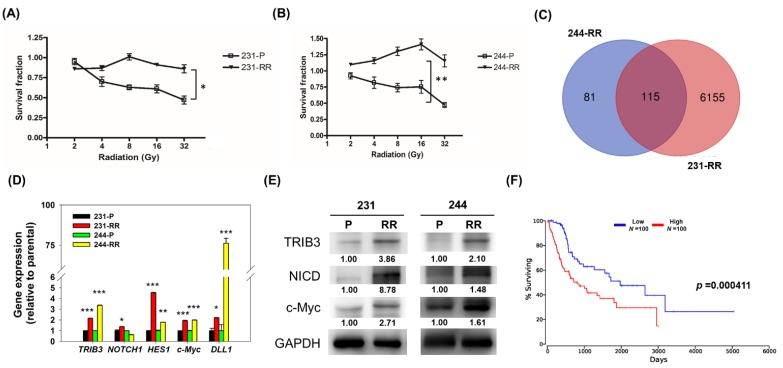
Tribbles pseudokinase 3 (TRIB3) expression and Notch1 activation were increased in radioresistant triple negative breast cancer (TNBC) cells. (**A**,**B**) MDA-MB-231, (**A**) AS-B244, (**B**) TBNC cells were repeatedly exposed to 2 Gy radiation for 10 cycles. The comparison of radiosensitivity between the parental TBNC cells (231-P or 244-P) and the derived lines after repeated radiation exposure (231-RR or 244-RR) was performed for 96 h in culture after accuminated radiation dosage as indicated with 3-(4,5-dimethylthiazol2-yl)-2,5-diphenyltetrazolium bromide (MTT) reagent. * *p* < 0.05; ** *p* < 0.01. (**C**) Total RNA was extracted from two TNBC cell lines as well as their derived radioresistant cells and microarray analysis of mRNA expression was performed. The lists of upregulated genes from two data sets were used for analysis of overlapping genes by the VennDiagram online tool (http://bioinformatics.psb.ugent.be/webtools/Venn/). (**D**) The mRNA expression of *TRIB3*, *NOTCH1*, *HES1*, *c-Myc*, and *DLL1* was determined by SYBR–Green based quantitative RT-PCR. * *p* < 0.05; ** *p* < 0.01; *** *p* < 0.001. (**E**) The protein expression of TRIB3, Notch intracellular domain (NICD), and c-Myc was analyzed by Western blot. (**F**) The prognostic analysis of TRIB3 in the overall survival among breast cancer patients was performed by the OncoLnc web-based analysis tool using the The Cancer Genome Atlas (TCGA) database (http://www.oncolnc.org/). The red line indicates high expression and the blue line indicates low expression.

In order to study the molecular changes in radioresistant TNBC cells, we first established radioresistant TNBC cells through repetitive exposure of 2 Gy radiation. After 10 cycles of 2 Gy radiation exposure, the surviving and continually proliferating TNBC cells from MDA-MB-231 (named 231-radioresistant, RR) or AS-B244 (named 244-RR) cells displayed a radioresistant feature up to 32 Gy (Figure 1A,B). We next purified total RNA from these two radioresistant TNBC cells and their parental counterparts and used microarray to explore the underlying molecular changes. There were 115 upregulated genes identified in both the 231-RR and 244-RR cells (Figure 1C) including *TRIB3* (the full lists of upregulated genes in 231-RR and 244-RR cells are provided in the Supplementary Materials). With the quantitative RT-PCR method, the expression of *TRIB3* was confirmed to be upregulated in these two radioresistant cells (Figure 1D). It has been reported that TRIB3 regulated Notch1 activation in lung cancer cells [13] and Notch1 activation is known to lead to radioresistance of TNBCs [14]. We next checked the mRNA expression of *NOTCH1*, its ligand, *DLL1*, and Notch target genes, such as *HES1* and *c-Myc*, by RT-PCR, and results indicated that Notch activation was enhanced in these two radioresistant TNBCs but without massively changing *NOTCH1* mRNA expression (Figure 1D). By Western blot, we further confirmed that the protein expression of TRIB3, the Notch intracellular domain (NICD), which is the activated form of Notch1, and c-Myc was upregulated in 231-RR or 244-RR radioresistant TNBC cells in comparison with their parental counterparts (Figure 1E). Analysis of The Cancer Genome Atlas (TCGA) data with the web-based OncoLnc analysis tool (http://www.oncolnc.org/) found that TRIB3 was an unfavorable prognostic factor in the overall survival of breast cancer patients (Figure 1F, *p* = 0.000411). From these results, it suggests that TRIB3 may contribute to the radioresistance of TNBCs.

### 2.2. Knockdown of TRIB3 in Radioresistant MDA-MB-231 Cells Reduced Notch1 Activation and Senisitized Cells Toward Radiation Treatment

**Figure 2:**
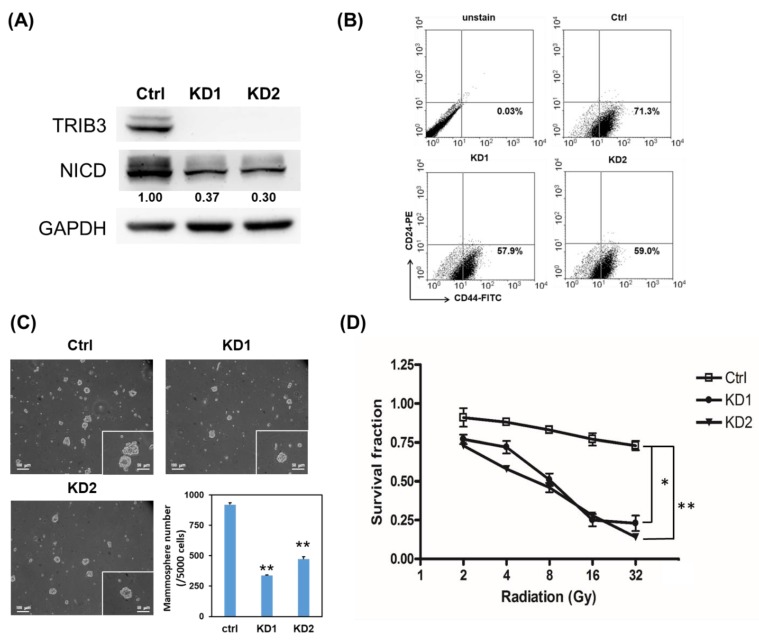
Knockdown of TRIB3 in radioresistant MDA-MB-231 cells reduced Notch1 activation and inhibited their cancer stem cell (CSC) activity. 231-RR cells were transduced with lentivirus carrying TRIB3 specific short hairpin RNAs (shRNAs) (KD1 or KD2) or control lentivirus (Ctrl, sh-LacZ) and selected for successfully transduced cells with 2 μg/mL puromycin for 48 h. (**A**) The expression of TRIB3 and the Notch intracellular domain (NICD) in transduced cells was determined by Western blot. (**B**) The CD24-CD44+ cells in transduced cells were determined by flow cytometric analysis. (**C**) CSC activity of transduced cells was examined by mammosphere formation assay. ** *p* < 0.01. (**D**) The transduced cells were irradiated with indicated dosage and cultured for 96 h. Cell viability was determined with 3-(4,5-dimethylthiazol2-yl)-2,5-diphenyltetrazolium bromide (MTT) reagent and survival fraction was calculated by setting the absorbance at a 570 nm wavelength of non-irradiated cells as 1.0. * *p* < 0.05; ** *p* < 0.01.

**Figure 3:**
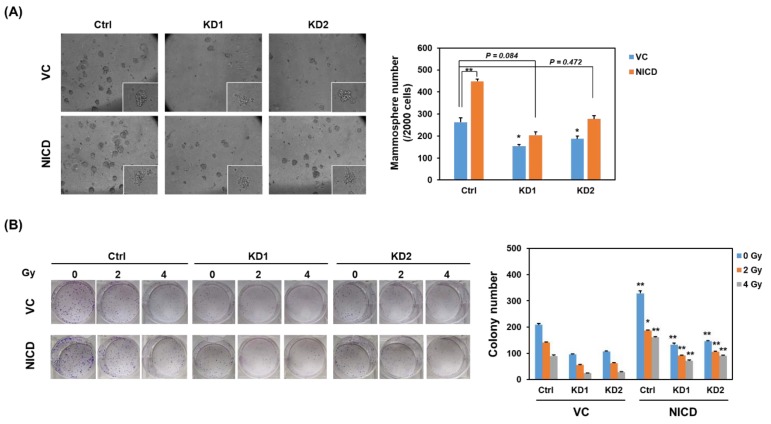
Forced expression of NICD reversed the inhibitory effect of TRIB3 knockdown in radioresistant TNBC cells. 231-RR cells were transfected with control vector (VC) or NICD plasmid (NICD) for 24 h followed by lentiviral transduction of TRIB3 specific shRNAs (KD1 or KD2) or sh-LacZ (Ctrl) for 48 h. (**A**) Cells performed mammosphere cultivation and formed mammospheres were counted at day 7. ** *p* < 0.01; * *p* < 0.05 when compared with the VC/Ctrl group. (**B**) Cells were seeded in 6 well-plates and received irradiation of 2 or 4 Gy. After 1-week cultivation, the formed colonies were stained by crystal violet and counted by Image J software. * *p* < 0.05; ** *p* < 0.01 when compared with VC groups of the same irradiation dosage.

Previous studies indicate that the development of radioresistance in breast cancer is associated with cancer stem cell (CSC) population [15,16]. We next examined if TRIB3 expression is associated with CSC population and self-renewal of radioresistant TNBC cells. With lentiviral delivery of TRIB3-specific short hairpin RNA (shRNA), we observed that knockdown of TRIB3 obviously reduced the NICD protein level in 231-RR cells (Figure 2A). It suggests that TRIB3 might mediate Notch1 activation in radioresistant TNBC cells. We further observed that the CD24-CD44^+^ CSC population (Figure 2B), as well as the mammosphere formation capability (Figure 2C) in 231-RR cells decreased after TRIB3 knockdown. We also examined the radiation sensitivity of 231-RR cells after knockdown of TRIB3, and the results showed that the loss of TRIB3 expression in 231-RR cells sensitized them toward radiation treatment (Figure 2D). In order to examine the role of Notch activation in TRIB3-mediated self-renewal or radioresistance in TNBC cells, the forced activation of Notch1 was conducted by transient transfection of NICD plasmid. The forced expression of NICD in 231-RR cells significantly increased the mammosphere number indicating the successful transfection (Figure 3A) and that the inhibitory effect of TRIB3 knockdown in mammosphere formation was reversed (Figure 3A). We also observed that the colonies of TRIB3 knockdown 231-RR cells after irradiation were significantly increased by forced expression of NICD (Figure 3B). These results suggest that TRIB3 is involved in CSC maintenance and radioresistance in TNBC cells through regulation of Notch1 activation.

### 2.3. Knockdown of TRIB3 Caused G1 Arrest in Radioresistant MDA-MB-231 Cells

**Figure 4:**
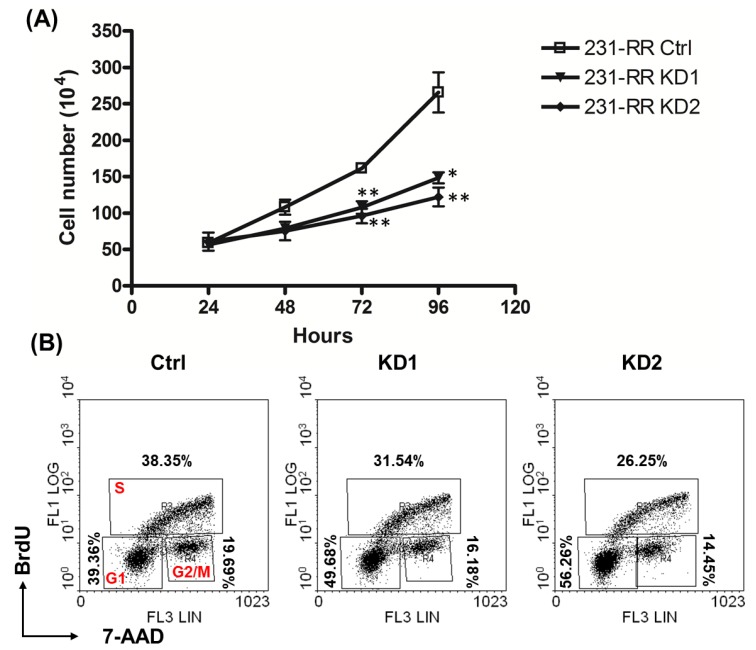
TRIB3 regulated cell growth and cell cycle progression in radioresistant MDA-MB-231 cells. Radioresistant MDA-MB-231 cells were transduced with lentivirus carrying TRIB3 specific shRNAs (KD1 or KD2) or control lentivirus (sh-LacZ, Ctrl) and selected for successfully transduced cells with 2 μg/ml puromycin for 48 h. (**A**) The selected cells were then harvested and plated into 12 well-plates at 1 × 10^5^ cells/well, and cells were harvested by trypsin/ ethylenediaminetetraacetic acid (EDTA) every 24 h until 96 h passed, followed by counting under inverted microscopy. *, *p* < 0.05; **, *p* < 0.01. (**B**) The selected cells were plated into 6-cm cell culture dishes at 3 × 10^5^ cells/dish and labeled with Bromodeoxyuridine (BrdU) at 46 h after plating. Cells were then harvested by trypsin/EDTA at 48 h and stained with BrdU incorporated cells as described in the Materials and Methods section. The percentage of each phase was calculated by WinMDI software.

We next checked if TRIB3 regulates cell growth in 231-RR cells. After TRIB3 knockdown, the cell growth of 231-RR cells became slower than those with transduction with control lentivirus (Figure 4A). We further checked the cell cycle progression of TRIB3 knockdown 231-RR cells, the results showed that the accumulation of cells in G_0_/G_1_ phase was observed in TRIB3 knockdown cells (Figure 4B). These data suggest that TRIB3 may regulate cell growth through controlling cell cycle progression in radioresistant TNBC cells.

### 2.4. TRIB3 Iinteracted with BCLAF1, BNIP1, and DDX5 in Radioresistant MDA-MB-231 Cells

**Figure 5:**
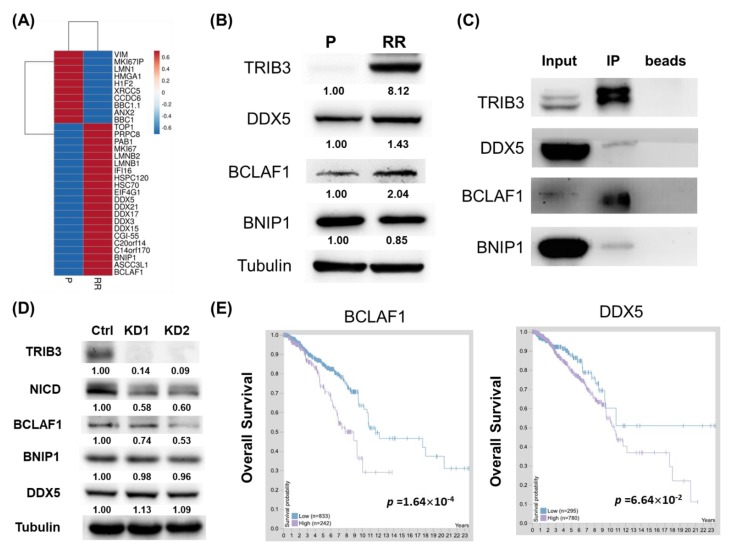
BCL2-associated transcription factor 1 (BCLAF1), BCL2 interacting protein 1 (BNIP1), or DEAD-box helicase 5 (DDX5) interacted with TRIB3 within radioresistant MDA-MB-231 cells. (**A**) Immunoprecipitation and mass spectrometry analysis were performed for total proteins from parental (P) or radioresistant (RR) MDA-MB-231 cells as described in the Materials and Methods section. The heatmap of identified proteins was created using the ClustVis web tool (https://biit.cs.ut.ee/clustvis/). (**B**) The expression of DDX5, BCLAF1, and BNIP1 between parental or radioresistant MDA-MB-231 cells was determined using Western blot. (**C**) The interaction between TRIB3 and DDX5, BCLAF1, or BNIP1 was determined by immunoprecipitation with anti-TRIB3 antibody and immunoblotting with antibodies of DDX5, BCLAF1, or BNIP1. (**D**) The expression of BCLAF1, BNIP1, or DDX5 in TRIB3 knockdown (KD1 or KD2) by lentiviral transduction of TRIB3 specific shRNAs or in sh-LacZ transduced (control) 231-RR cells was determined by Western blot. (**E**) The prognostic analysis of BCLAF1 or DDX5 in overall survival among breast cancer patients was obtained from The Human Protein Atlas website (https://www.proteinatlas.org/).

Since TRIB3 is known to function as a protein scaffold within cells, we were next interested in the interactome of TRIB3 in radioresistant TNBC cells. After immunoprecipitation of TRIB3 from 231-P or 231-RR cells, the precipitated proteins were further analyzed by mass spectrometry and the results are shown in Figure 5A. Among the interacting proteins with increased features in radioresistant cells, a group of DEAD-box RNA helicases (DDXs) was observed (DDX3, DDX5, DDX15, DDX17, DDX21) and the DDX5 was the most abundant among them. With Western blot analysis, the expression of DDX5 was increased in 231-RR cells in comparison to 231-P cells (Figure 5B). Bcl-2-associated transcription factor 1 (BCLAF1), a protein originally identified as a protein partner for the adenoviral bcl-2 homologue E1B19K [17], also displayed an upregulated expression pattern in 231-RR cells (Figure 5B). We next tried to confirm these three putative TRIB3 interacting proteins including BCLAF1, DDX5, and BCL2 interacting protein 1 (BNIP1) with immunoprecipitation and Western blot analysis. As shown in Figure 5C, all three proteins were co-precipitated with TRIB3 in 231-RR cells. We also examined the expression of these three TRIB3 interacting proteins after knockdown of TRIB3 and the results revealed that only the expression of BCLAF1 decreased in TRIB3 knockdown 231-RR cells (Figure 5D). Based on the data of The Human Protein Atlas, BCLAF1 and DDX5 had an unfavorable prognostic factor among breast cancer patients, although only BCLAF1 reached statistical significance (Figure 5E). With the web resource of UALCAN [18], the mRNA expression of BNIP1 (Figure S1, *p* = 0.048) and BCLAF1 (Figure S2, *p* = 0.00014) displayed a significantly negative correlation with breast cancer patients’ overall survival. These data suggest that TRIB3 may interact with BCLAF1/BNIP1/DDX5 to play a pathogenic role in breast cancers.

## 3. Discussion

In the present study, we discovered that TRIB3 expression was induced in radioresistant TNBC cells and the knockdown of TRIB3 in radioresistant TNBC cells could sensitize cells toward radiation treatment (Figure 2D), which may correlate with the inhibition of the Notch1 activation after TRIB3 knockdown (Figure 2A). It is known that radiation can induce Notch1 activation in breast CSCs [19] and non-small cell lung cancer cells [20] to confer radioresistance. Radiation can induce the expression of Notch isoforms and several Notch ligands in breast CSCs, and the treatment of a γ-secretase inhibitor, the Notch inhibitor, prevented the radiation induced increases in breast CSCs [19]. These studies suggest that to target Notch activation could be a promising strategy in cancer radiosensitization. Izrailit et al. [21] have reported that TRIB3 was a master regulator of Notch through the mitogen activated protein kinase (MAPK)-extracellular signal–regulated kinase (ERK) and TGF-β pathways in breast cancer cells. The expression of jagged 1 (JAG1), one of the Notch ligands, was inhibited with knockdown of TRIB3 in breast cancer cells [21]. We also observed that one of the Notch ligands’ (DLL1) expression was elevated in both 231-RR and 244-RR cells at the mRNA level (Figure 1D). It suggests that the increased Notch1 activation may be due to the upregulated Notch ligands by TRIB3 in radioresistant TNBC cells. Wennemers et al. [12] have demonstrated that the expression of TRIB3 was associated with hypoxia regions in MDA-MB-231 xenograft tumors and decreased the cell survival under hypoxia condition. Tumor hypoxia is a known mechanism in cancers to acquire radioresistance [22,23]. It should be worthy to investigate the involvement of hypoxia in the upregulation TRIB3 in radioresistant TNBC cells. On the other hand, TRIB3 has been suggested as a sensor of tumor microenvironment [24]. Nutrient deprivation or the induction of endoplasmic reticulum stress could upregulate TRIB3 expression in basal-like breast cancer through ubiquitin specific peptidase 9 X-linked (USP9x)-mediated deubiquitination and stabilization of TRIB3 [24]. Furthermore, USP9x could also be required for cellular stress-induced Notch activation through stimulation of JAG1 expression [24]. It is also interesting to further investigate if USP9x is involved in TRIB3 upregulation and Notch1 activation in radioresistant TNBC cells.

Yu et al. [25] have demonstrated that inhibition of TRIB3 expression in HaCaT cells resulted in decreased cell growth and increased G_0_/G_1_ population. We also observed similar phenomena in radioresistant MDA-MB-231 cells (Figure 4). Xu et al. [26] have observed that TRIB3 interacts with carboxy-terminal binding protein (CtBP)-interacting protein (CtIP) in the nuclei of HeLa cells under UV stimulation. You et al. [27] found that CtIP could sense double-strand breaks (DSBs) and were recruited to DSBs to regulate ataxia-telangiectasia mutated (ATM) kinase activity. It has been demonstrated that glioblastoma stem cells (GSCs) displayed a radioresistant feature which was associated with increased ATM activation [28]. The inhibition of ATM activation achieved a great radiosensitizing effect in GSCs [29]. Taken together, we hypothesize that TRIB3 may be involved in radiation-induced DNA damage in TNBC cells through CtIP/ATM pathways, but it remains to be experimentally proved in the future.

We found that BCLAF1 could be one of the possible interacting proteins within radioresistant TNBC cells. Lee et al. [30] previously demonstrated that BCLAF1 was a radiation-induced H2AX-interacting protein and regulated apoptosis and DNA repair of cells which had received radiation. Actually, BCLAF1 is known as an RNA processing factor and Vohhodina et al. [31] found that BCLAF1 selectively regulated mRNA splicing and export of transcripts encoding DNA damage response proteins including ATM. Furthermore, BCLAF1 has been found in DNA damage-induced BRCA1 protein complex [32]. Knockdown of BCLAF1 in MCF7 breast cancer cells enhanced radiation-induced cell death suggesting that BCLAF1 could promote resistance to radiation-induced DNA damage [32]. Although BCLAF1 was suggested as a tumor suppressor by Lee et al. [30], it is clear that it is a poor prognostic factor in breast cancer patients at both protein and mRNA levels (Figure 5E and Figure S1) and BCLAF1 expression in TNBC cells are potentially regulated by TRIB3 expression (Figure 5D). It is required to further investigate the role of BCLAF1 in TRIB3-mediated radioresistance in TNBC cells.

In our mass spectrometry data, DDXs were a group of proteins with TRIB3 interacting potential (Figure 5A). The expression of DDX3 has been reported to be increased in lung cancers, and inhibition of DDX3 by RNA interference or a small molecule inhibitor resulted in suppression of Wnt signaling and led to tumor regression [33]. In MDA-MB-231 cells, Wang et al. [34] found that knockdown of DDX5 resulted in inhibition of cell proliferation and suppression of oncogenic microRNA expression including miR-21 and miR-182. Recently, Li et al. [35] reported that downregulation of miR-21 inhibited radioresistant phenotypes of esophageal squamous cell carcinoma. The DDX5 protein also regulates the expression of genes encoding DNA replication factors in breast cancer cells [36] and has been suggested to play a role in the responses to chemotherapy and radiotherapy [37]. The role of DDX5 in TRIB3-mediated radioresistance of TNBC cells requires further investigation.

## 4. Materials and Methods

### 4.1. Cell Culture and Mammosphere Cultivation

The TNBC cell line, AS-B244, derived from BC0244 xenograft human breast cancer cells were established by Alice L. Yu’s laboratory (Institute of Stem Cell and Translational Cancer Research, Chang Gung Memorial Hospital at Linkou and Chang Gung University, Taoyuan, Taiwan) [38] and were cultured in minimal essential media (MEM)-α medium (Invitrogen, Thermo Fisher Scientific, Waltham, MA, USA) supplemented with 10% fetal bovine serum (Biological Industries, Cromwell, CT, USA), 0.1 mg/mL bovine insulin (Sigma–Aldrich, St. Louis, MO, USA), 1 mM sodium pyruvate (Invitrogen), and 2 mM Glutamax (Invitrogen). The cells were maintained in a 5% CO_2_ air humidified at 37 °C. The TNBC cell line, MDA-MB-231, was obtained from ATCC (Manassas, VA, USA) and cultures as per ATCC’s recommendations. For mammosphere cultivation, cells were suspended in Dulbecco’s modification of Eagle medium (DMEM)/F12 medium containing 0.4% bovine serum albumin (Sigma–Aldrich), 20 ng/ml epidermal growth factor (PeproTech Asia, Rehovot, Israel), 20 ng/mL basic fibroblast growth factor (PeproTech), 5 μg/mL insulin, 0.5 X B27 supplement (Invitrogen), and 4μg/ml heparin (Sigma–Aldrich) followed by seeding into an ultralow attachment 6-well plate (Corning Inc., Corning, NY, USA) at a density of 5000 cells/well or 2000 cells/well. The formed mammospheres were counted under inverted light microscopy.

### 4.2. Establishment of Radioresistant Triple Negative Breast Cancer Cells 

The TNBC cells were seeded into 6-cm culture dishes and exposed to irradiation dosages of 2 Gy by Elekta Axesse^TM^ linear accelerator (Elekta AB, Stockholm, Sweden) at a dose rate of 6 Gy min-1 when cells reached to 80% confluency. After irradiation, the dishes were replaced with fresh media and the cells were cultured at 37 °C incubator. After the cultured cells reached 80% confluency again, the cells were sent for next round of 2 Gy irradiation. The radiosensitivity of TNBC cells were determined when cells received a total of 20 Gy radiation dosage by sequential irradiation (2, 4, 8, 16, 32 Gy). The survival fraction of cells was determined at 72 h post-radiation by MTT reagent to determine the absorbance at 570 nm wavelength or trypan blue exclusion assay under a cell counting chamber. 

### 4.3. Quantitative Real Time-PCR

Total RNA was extracted and purified by RNA extraction kit (Zymo Research, Irvine, CA, USA) and cDNA were synthesized with first strand cDNA synthesis kit (Fermentas Inc., Waltham, MA, USA). Expression of genes were detected with specific primers and KAPA SYBR^TM^ fast qPCR kit (Kapa Biosystems, Inc., Wilmington, MA, USA) with an ABI StepOnePlus™ Real-Time PCR System and analyzed with the StepOne software v2.3 (Applied Biosystems, Life Technologies Corp., Carlsbad, CA, USA). Fifty nanograms of cDNA sample were used in a SYBR Green-based qPCR reaction; the cycling conditions were as follows: 50 °C for 2 min, 95 °C for 10 min, followed by 40 cycles of 95 °C for 10 s, and 60 °C for 1 min. The end-point used in the real-time quantification was calculated by the StepOne software, and the threshold cycle number (Ct value) for each analyzed sample was calculated. Each target gene was normalized to glyceraldehyde-3-phosphate dehydrogenase (GAPDH) to derive the change in Ct value (△Ct). The changes of genes between parental TNBC cell lines and the radioresistant sub-lines were calculated by 2^−△△Ct^. The primer sets used in this study are listed as followed: TRIB3-F:5′-ACCGTATCCCTGAGCCTGA-3′, TRIB3-R:5′-CTTGTCCCACAGGGAATCAT-3′, Notch1-F: 5′-ACTGTGAGGACCTGGTGGAC-3′, Notch1-R:5′-TTGTAGGTGTTGGGGAGGTC-3′, Hes1-F:5′-CGGACATTCTGGAAATGACA-3′, Hes1-R:5′-CATTGATCTGGGTCATGCAG-3′, c-Myc-F:5′-AATGAAAAGGCCCCCAAGGTAGTTATCC-3′, c-Myc-R: GTCGTTTCCGCAACAAGTCCTCTTC-3′, GAPDH-F:5′-CAATGACCCCTTCATTGACC-3′, GAPDH-R:5′-TGGACTCCACGACGTACTCA-3′.

### 4.4. Western Blot Analysis

Cells were washed with 1X PBS and lysed in radioimmunoprecipitation assay (RIPA) lysis buffer (GeneTex Inc., Hsinchu City, Taiwan). Twenty-five μg of extracted protein was separated by SDS-PAGE and transferred to the polyvinylidene difluoride (PVDF) membrane (Immobilon-P, Merckmillipore, Danvers, MA, USA). The membrane was then incubated with primary antibodies at 4 °C overnight. After washing with Tris-buffered saline (TBS)/0.05% Tween-20, the membrane was then incubated with specific secondary antibody conjugated with horseradish peroxidase (HRP) at room temperature for one hour. The signals were then developed by incubation with chemiluminescence substrate (PerkinElmer Inc.) and captured with the Luminescence-Image Analyzer (FUSION SOLO, Vilber Lourmat Deutschland GmbH, Germany). The antibodies used in this study were listed as followed: rabbit IgG anti-TRIB3 was purchased from Proteintech Group, Inc. (Rosemont, IL, USA); rabbit IgG anti-Notch1 was purchased from Abcam (Cambridge, UK); rabbit IgG anti-c-Myc antibody was purchased from GeneTex Inc. Mouse IgG anti-BCLAF1, mouse IgG anti-BNIP1, and mouse IgG anti-DDX5 antibodies were purchased from Santa Cruz Biotechnologies, Inc. (Dallas, TX, USA). Horseradish peroxidase-conjugated anti-rabbit or anti-mouse IgG secondary antibodies were purchased from Cell Signaling Technology, Inc. (Boston, MA, USA).

### 4.5. Lentivirus-Mediated Short Hairpin RNA Delivery

The TRIB3 (TRCN0000307989 and TRCN0000196756) or LacZ (TRCN0000231722) specific shRNA lentiviral vectors were obtained from the National RNAi Core Facility at the Institute of Molecular Biology, Academia Sinica (Taipei, Taiwan). Lentivirus production and transduction into TNBC cells were performed as described by our previous report [39]. 

### 4.6. Transfection of Notch Intracellular Domain Expressing Plasmid

The expression vector of NICD and a negative control vector were purchased from BPS Bioscience, Inc. (Cat. No. 79503, San Diego, CA, USA). The transfection was performed with jetPrime^TM^ transfection reagent (Polyplus transfection, Illkirch, France). Briefly, 2 × 10^5^ cells were seeded into 6-well plates overnight. On the day of transfection, 2 μg of plasmid DNA was first diluted in 200 μl jetPrime^TM^ buffer and complexed with 4 μl jetPrimeTM transfection reagent at room temperature for 15 min. The DNA:reagent complexes were then added into wells and incubated at 37 °C CO_2_ incubator for 6 h followed by changing to fresh culture medium. Transfected cells were used for further experiments after 24 h.

### 4.7. Cell Growth and Cell Cycle Analysis

For analysis of cell growth curve, cells were seeded into 12-well plates at the initial density of 2 × 10^4^ cells/well. The cells were then harvested every 24 h until 96 h and cell numbers were counted by trypan blue exclusion assay. For cell cycle analysis, cells were seeded into 6-cm dishes at the initial density of 2 × 10^5^ cells/dish. Before cell harvesting, 10 μM BrdU was added to dishes and incubated at 37 °C for two h. The harvested cells were then fixed, permeabilized, and incubated with anti-BrdU- fluorescein isothiocyanate (FITC) antibody according the manufacturers’ protocol (BD Biosciences, San Jose, CA, USA). Before fluorescence-activated cell sorting (FACS) analysis, the cells were stained with 7-aminoactinomycin D (7-ADD) (BD Biosciences) on ice for 10 min. The fluorescence signals were detected by Epics XL flow cytometry (Beckman Coulter, Inc., Atlanta, GA, USA) and data were analyzed by WinMDI software (J. Trotter, Scripps Research, La Jolla, CA, USA).

### 4.8. Mass Spectrometry Analysis of TRIB3 Interacting Proteins

Immunoprecipitation (IP) was performed by incubation of 1 μg anti-TRIB3 antibody with 1 mg total protein prepared from MDA-MB-231 cells and the radioresistant sub-line at 4 °C for overnight followed by the incubation with Protein A conjugated magnetic beads (GE) at RT for one hour. The IP products were applied to 10% SDS-PAGE for separation. After electrophoresis, the gel was subsequently visualized through Coomassie blue staining and were sliced into 10 small gel pieces (<1 mm^3^), followed by in-gel digestion. Briefly, the procedure of in-gel digestion included the following steps in order: (a) de-staining with 50% acetonitrile (ACN) and 25 mM ammonium bicarbonate (ABC); (b) reduction using freshly prepared 10 mM dithiothreitol for 45 min at 58OC; (c) alkylation using freshly prepared 55 mM iodoacetamide for 45 min at room temperature in the dark; (d) enzyme digestion with 3 ng/µL sequencing grade trypsin in 25 mM ABC solution at 37OC for 16–18 h; (e) extraction of tryptic peptides using a 60% ACN/1% trifluoroacetic acid solution. After drying to remove the solvent, the tryptic peptides were analyzed by mass spectrometer (MS) analysis. The full-scan survey MS experiments (m/z 320–2000) were performed in linear ion trap-Fourier transform ion cyclotron resonance mass spectrometer (LTQ-FTICR MS) with a mass resolution of 100,000 at m/z 400. The top ten most abundant multiply charged ions were sequentially isolated for MS/MS by LTQ. The following MASCOT search parameter settings were used: peptide tolerance was 5 ppm with 2+ and 3+ peptide charges and MS/MS tolerance was 0.5 Da. Two missed cleavages by trypsin were allowed, carbamidomethyl (C) was used as a fixed modification and oxidation (M) and deamidated (NQ) were used as variable modifications. The significance threshold for the identification was set to *p* < 0.01. Label-free quantitative analysis was performed using quantitative proteomics software (MaxQuant; Max Planck Institute of Biochemistry, Munich, Germany) [40]. 

### 4.9. Analysis of the Correlation between TRIB3/BCLAF1/BNIP1/DDX5 and Breast Cancer Patients’ Survival 

The correlations between TRIB3/BCLAF1/BNIP1/DDX5 and breast cancer patients’ survival were analyzed by Oncolnc, The Human Protein Atlas (https://www.proteinatlas.org/), or UALCAN (http://ualcan.path.edu/index.html) web-based analysis tools. The database used by Oncolnc or UALCAN was obtained from The Cancer Genome Atlas.

## 5. Conclusions

In the present study, we found that TRIB3 expression, as well as Notch1 activation, was increased in TNBC cells which achieved a radioresistant phenotype. Inhibition of TRIB3 expression suppressed Notch activation and sensitized radioresistant TNBC cells toward radiation treatment. The expression of TRIB3 was also involved in cell proliferation and cell cycle progression of radioresistant TNBC cells. With mass spectrometry and immunoprecipitation analysis, three potential TRIB3-interacting proteins were identified and two of them, BCLAF1 and DDX5, served as poor prognostic factors in breast cancer patients at the protein level. Our data suggest that TRIB3 expression may serve as a biomarker for prediction of radiation response in breast cancer patients and targeting TRIB3 could be a potential strategy for developing future breast cancer therapy.

## Supplementary Materials

The following are available online at http://www.mdpi.com/2072-6694/11/2/127/s1, Gene lists: Full lists of upregulated genes in radioresistant TNBC cells, Figure S1: The correlation of BNIP1 mRNA expression on breast cancer patients’ overall survival, Figure S2: The correlation of BCLAF1 mRNA expression on breast cancer patients’ overall survival.
