# Supplementary material for: Tribbles Homolog 3 Involved in Radiation Response of Triple Negative Breast Cancer Cells by Regulating Notch1 Activation
Source: Cancers (Basel). 2019 Jan 22;11(2):127. doi: 10.3390/cancers11020127 (PMC6406679; doi:10.3390/cancers11020127)
Supplement: Supplementary file 1 [file cancers-11-00127-s001.zip › Full list of upregulated genes in radioresistant TNBC cells.pdf]

Calculate and draw custom Venn diagrams

Input files:

List names	number of elements	number of unique e
AS-B244	206	196
MDA-MB-231	7101	6270
**Overall number of unique elements**		**6351**

Image result:

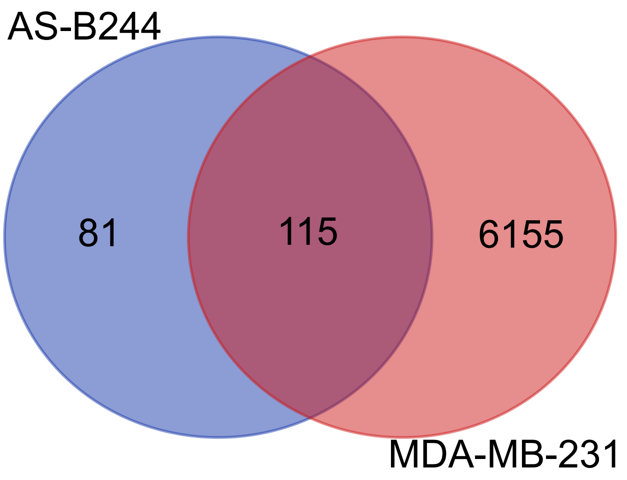

Save

Text results:

Save text

Names	total	elements
AS-B244 MDA-MB-231	115	LOC100653071|CRTAP TRIB3 ANXA3 CRELD2 SLC22A14 PHGDH GADD45B AKT2 EGFR PACS1 SLC3A2 BHLHE40 GPR56 LOC101060798 PKP3 IGFE<br>PPAP2C ZMYND8 PACS2 NUPR1 NLGN2 CC2D1A PLS1 HSPA1A SFN KEAP1 KRI1 TNFRSF12A FOSL2 MAP2K2|LOC407835 TRIM16|TRIM16L DDIT4 V<br>SLC38A1 ZMIZ1 AKR1C3 SEPHS2 PDIA4 SESN2 AKR1C1|LOC101060798 GOLGA4 GARS KRT18|KRT18P55 SLC38A10 HYOU1 PERP FN1 LAMB3 ZFF<br>OSGIN1 AQP3 PMAIP1 DNM2 TMEM74B CHCHD10 SAA1 KRT7 LOXL4 SLC7A1 ZFAND2A EPAS1 MAP1LC3B2 PLAUR VAMP8 PHLDA3 RAB6A|WTH3D<br>HERPUD1 SRXN1 SLC7A5 AK4 DDIT3 WNT10A TRRAP DST HSPA1B SYVN1 F11R ATP2A2 KCNN4 PNPLA2 SLC39A6
AS-B244	81	CXCL3 SHBG FAM110C TACR1 UBN2 BST1 KRT15 MANF GPX2 EPB41L3 VEGFA TBC1D24 KRT9 PPP5C NTRK2 OR2H2 ESRP1 SDF2L1 LTBP3 C9orf<br>SERPINE2 DMKN NFIX KLK5 ASNS KLK10 KRT6B PPFIA4 MFHAS1 SCN2B C1orf172 S100A9 TRIM74|TRIM73 KRT17 PPP1R1B EPS8L3 LGALS7B|LGA<br>CHODL-AS1 TFCP2L1 ZXDA S100A14 ZRANB1 TNNI2 SLPI HSP90B1|HSP90B2P INHBB FUZ TNC HSPA6|HSPA7 UBE2M|UBE2MP1 HSPA5 LOC10013
MDA-MB-231	6155	MSRB1 DPM2 AACS FSTL1 CREB3L1 RPS11 PNMA1 CD44 KRTCAP2 FARP1 TMEM216 HAUS2 ITGA5 NDUFS6 TUT1 ERAL1 ECHS1 VPS4A ABCB7 P<br>LOC401397 BRIX1 TMSB10 LMAN1 ESYT1 LCORL RAB1B GALNT3 CHD8 SUMO1 MSL1 UBAC1 DDB1 UQCR11 MYO9B TOP1MT CCNI 11-Sep GPSM2<br>MIER1 CHD9 DBNDD2|SYS1-DBNDD2 SEMA4D MMS22L AGPAT1 MET THRAP3 LYAR INIP GSTCD COPS7A MAX OSBPL8 PROSC NOC2L|LOC401010<br>SLC25A14 UBA1 WDR34 ATXN10 ECH1 MAPKAPK2 FAHD1 LHFPL2 EPT1 DHRS11 CCDC34<br>TBC1D3G|TBC1D3B|LOC101060303|LOC101060287|TBC1D3|TBC1D3F|TBC1D3C|LOC101060506|LOC101060489|LOC101060440|LOC101060421|LOC1<br>TAOK3 STK25 ITGA3 BRCA1 KIAA1033 CDC25B TMEM19 STX8 LUZP1 SLK EIF4G3 NOL8 GRPEL1 BAZ1B LMNB2 ROCK2 PAM16|CORO7-PAM16 7-Ma<br>ATL2 PDCD5 DGUOK AP1S2 RAB33B ALDOC RBM17 ZNF212 NUDT1 MANEA ACBD3 ZNF83 KIF4A|KIF4B SUPT4H1 CEP350 SMYD3 CST7 RAPGEF6<br>RPIA|LOC101060545 FAM98A EXOC3 RAD23B NCOR2 MED27|CRSP8P C19orf43 CTDSP2 CCNY DUSP4 SSU72 TIAL1 FOXM1 HIST1H1E SMUG1 OST<br>ING5 F8A1 LOC642852 ALG2 PLEKHG4 PPP1R3D GRB2 NBPF15|NBPF11|NBPF8|LOC100288142|NBPF24|NBPF16|LOC101060226|LOC100996575 F2F<br>TUBB RARRES3 GAS2L1 TOR3A KIAA0100 PMF1 PREP HS2ST1 CD82 PMM1 LCMT1 EIF1AX GCLM PPM1A ARL13B CAMTA1 ZNF239 PTP4A2 IL24 S<br>TOMM34 CARD8 C9orf16 ERBB2IP FUS IMMP1L RSRC1 CLCN4 AURKAPS1|AURKA PTRF CHST11 PEX11B|LOC101060567 MMP15 PWP2 MTMR14 PS<br>CEP120 ZNHIT2 RICTOR SPTBN1 IRS1 LSM4 CDC42EP3 TMEM104 HSF2 SAMM50 SRP72 MFN2 POMT1 NUCB1 MTX1 LRRC8A OGFOD2 BRK1 CAPI<br>SARS KDM2A MAN2B1 OFD1 PRDX4 ABHD12 PDP1 ATF7 ABCF2 KDM4B GNG12 HABP4 C17orf85 NSUN5P2|NSUN5P1|NSUN5 LOC101060179 SPG2<br>ZNF655 TMEM11 MRPL15 ARAP1 NQO2 AGPS RPUSD3 NEU1 UBE2L6 PIN1P1 MED14 HP1BP3 IPO13 MAK16 PCCB UNG TSN SPRY2 XPC KLHL7 CC<br>NOP58 RPRD1B CHMP7 SERF1A|SERF1B FKBP4 PTPN2 DOM3Z SF3A3 SLC25A51|SLC25A52|SLC25A51P1|LOC494141 AHI1 KIF14 FAM175A YIPF2 I<br>RPL21|RPL21P28|RPL21P44 MAD2L1 EIF4A3 ATP6V1C1 CHMP2B CAMSAP1 ACO2 RPS21 RBM6 COQ10B PSMF1 SORBS2 UBE2N CYSTM1 NFE2L2<br>POLR2J3|UPK3BL|POLR2J2 IDS PARG C1orf112 DYRK4 CXCR7 MICALL1 UIMC1 GOT2 WBSCR16|LOC653375 NFYC LOC101060301|LOC649330|HNR<br>CIDECP|CIDEC BRD4 ZNF146 POLRMT VMP1 C19orf6 NDUFA13 CD151 ARFRP1 MRPL41 NENF MTG1 SCAMP3 TTC13 ENTPD7 SOX9 CASC4 CUL4E<br>TNKS2 TMEM241 MRTO4 C1QBP NDUFB8 TMEM248 R3HDM2 PAQR3 TMED7 TRAM2 FST ARHGEF26 PAWR TLK1 TIGD2 MTMR12 RELL1 LDLR SMF<br>FAM160B1 GPATCH8 EIF3D KIAA0930 HPS1 MPC2 KIF4A RNF7 TFE3 ZNF511 PGM1 POLR3G CPD SNRPC KCTD13 TANK RCAN1 IL8 IRF7 PELI3 SET<br>PIGG TM9SF4 ZNF264 TAF9 TERF2 RAB5A TNFRSF1A METTL5 MSTO1|MSTO2P ACADVL ISCU GTF2H5 CECR5 ICA1L XIAP GPS1 BDP1 AKR1C3|AK<br>IMPAD1 MGC57346 DAGLB HCFC1R1 LOC101060570 GLIPR2 SEPHS1 EHD2 SPG7 ZMYM6NB SAT2 MCM10 TCF19 RMDN3 C6orf52 NUP133 KIAA075<br>NT5E PARP4 FAM129B SAFB2 IL18 BEGAIN NCK2 OXA1L ADD1 PSMD12 ANP32A TRPC1 ACLY MEX3D ERCC8 CHCHD5 SMS NFAT5 NUDT22 RRAG<br>TMEM106C CPA4 RPL39 HERC3 BTBD10 ZBTB47 MELK RABGGTB PODXL SPATS2L FAM122B CDK16 GEMIN6 HRAS BSDC1 RABL6 METTL16 ACOT<br>SOCS4 PSMB8 HN1 XRCC1 SDR39U1 PPP6R2 ELL CDH11 ZBTB41 AHCTF1 CDS2 AP2S1 C2orf69 VAT1 SENP3 FAM213A CCDC14 NSUN5 TUFM SF3<br>SUPT16H PHYH TOMM22 MUS81 SOCS3 UNKL ZNF839 POP4 OIP5 PPAPDC1B IL10RB TMEM132A ARHGAP10 CGRRF1 RUVBL2 MCAT ARID1B RPL<br>SRSF3 ICAM2 SH3GL1 SLC25A39 GSK3B PDXP KIF1B RALB GPD1L VPS37B C6orf120 TNFAIP3 PSRC1 PIGY|PYURF PIGT ALAS1 ATG3 C17orf62 MR<br>GLTPD2 TESK1 FBXO7 SFXN3 TSEN54 CA12 ZNF3 LRRFIP1 PPP4R1 GCLC CBX2 ZC3H14 RBMXL1 HYPK|SERF2-C15ORF63 UPF1 CTDP1 ZSWIM4 S<br>GALNT10 PQLC3 GPR110 MKKS STK39 ARL17B|LOC100996709 ACOX3 SMURF2 BANF1 RPS6KA2 EBP HMBS SLC25A24 TOR1A ACER3 PES1 UCP2<br>FBRS NDUFB3 FPR1 FMNL1 DEPDC1 KIF23 ATL1 PHOSPHO2-KLHL23|KLHL23 FAM83A USP38 ZDHHC17 ARMCX3 ARHGDIB MAGEF1 NDE1 QSOX2<br>NBPF3|LOC100288142|LOC101060202|NBPF11|NBPF8|LOC100996575|NBPF24|NBPF15|NBPF12|LOC101060226|LOC101060238|NBPF16 PTPN23 FAM<br>AP1S3 UCKL1 PAM OBSL1 SLC12A7 RBM4B THAP4 FAM203B|FAM203A OGFRL1 SLC39A4 MDC1 LINC00493 STARD8 FNDC4 OCEL1 ATP11B FBXO3<br>URGCP ZFAND1 AXL RINT1 MAF1 CD276 SPAG5 KRT80 SRP14 ACADM XPO6 CCDC117 SRSF10|LOC100996657 C6orf89 DUSP3 FAM8A1 C7orf50 RT<br>HAUS1 RAD23A MT1E MZT2B|MZT2A ABCC6|ABCC6P2 ELFN2 IRAK2 SCAMP4 GHITM NDUFB7 TXLNG FOXN3 CCDC3 MIER2 PNMA2 SH3BP2 AGRN

ATPIF1 PDLIM1 APBB3 UQCR10 WDFY3 RNF126 GPSM1 CLIC4 PSMB7 MESDC1 SHQ1 C5orf51 TNK1 THUMPD3 ELP6 POLR1B TACC1 SAMHD1 TRI
SPANXC|SPANXF1|SPANXA2|SPANXA1|SPANXB1|SPANXB2|SPANXE|SPANXD SDF4 SNORD110 TAP2 XKR8|RNF216P1 KLHDC3 C19orf70 EFHD2 CX
SNX5 METTL6 MAPKAPK3 SOD1 C12orf5 AFAP1 MSRB2 CAPN2 ZFP36L1 TMX3 SEC22C FXYD5 TWISTNB CCT6P1|CCT6P3|CCT6A NKX3-1 CHD2 HI
ERBB2 CREB1 ABCB9 NUDT4|NUDT4P1 LOC101060404|LOC100132999|LOC101060562|LOC101060722|LOC101060698|LOC642441|LOC101060645|L
RPS3A MXRA7 CFL2 DBI BTBD1 ABCF1 CDT1 FCF1 RFFL|RAD51L3-RFFL APOOL SMARCA4 DIABLO ARMC9 RPS6KA4 LRP8 MRPL14 CENPW AGL
CLPTM1L TTL EML4 SGTA OBFC1 VIMP PRPF8 SSB SKI PLEKHB2 TIMM23 OAS2 RPL28 ZNF544 TRIM11 D2HGDH TMEM50B DEGS1 HM13 FAIM TBL
TOM1L1 TMEM167A BCAT2 MFN1 RTCA BCKDHA RAB4A C11orf80 FAM134C SNX11 THBS1 APOO FARSA CAMSAP2 ARMCX1 ACSL1 ABL1 PTPRG C
LRRC26 CCNE2 CDC45 CRIP2 TAZ CWC22 DAXX FUT11 C16orf72 IGF2|INS-IGF2 USP33 PPP1CB C15orf39 ABHD8 MPLKIP TAF11 PAAF1 CCDC136 C
NCOA1 GUF1 CDK7 WDR44 HRH1 CRELD1 NUDT16L1 RNPEP MCM2 LRRC37B|LRRC37BP1 RBMX2 KIAA0922 SYF2 ZBTB10 FBXL17 CEP192 RPL9
LRSAM1 B3GALT6 HDAC11 KIAA1731 FAM50B MTO1 DHX29 HADHB ADAR PWP1 BAHD1 PLXNB2 SLC15A3 UQCRH|UQCRHL METAP2 NUMB RHOQ
CANT1 UNC50 C21orf33 CDC37 RUNX2 BRE SRPX PPP2CA LRP12 POLR2A ODC1 SDC3 OGFOD1 ADORA2B NR2F6 TMEM218 MSMO1 ZFYVE16 KR
HIP1R PLEKHA2 TMUB1 L3HYPDH TVP23B|TVP23C EMC6 LRRC1 TOMM70A ZNF559 NT5C3 TNFRSF18 SLC35G2 HERC2P9|HERC2P2 DET1 GALT T
ICAM3 TM9SF3 ADAM8 BCL7B MAN2B2 PPP6R3 RNH1 BICD2 PTTG3P ALKBH5 ARL6IP4 TOMM5 GPR89A|GPR89C|GPR89B JUP TMEM41A RAB40C
MYL9 CDC23 GUCD1 TAB3 EHD1 KIAA1586 C9orf69 RAB11FIP5 NCK1 CDKN1A CHCHD6 SLC2A4RG FBXL3 DPH1|OVCA2 LSR MIS12 CUL7 EIF2B1 G
STRA13 HIST1H4H RPP25 CCDC71L TMEM133 PTPMT1 AJUBA TAF1C HOXC11 CIAPIN1 ZMIZ2 SLC46A3 PTPRJ CSTB PTRH2 ZNF668 HILPDA C22o
TMSB15B|LOC101060235 CCNF KLHL5 SRM RPP25L MRPS6 ANKRD33B TCEAL3|TCEAL6 AKAP8 WASH1|WASH3P|LOC100288778|WASH7P|WASH2
SMARCAD1 ATG12 FNDC3B RNMT FTSJD2 VMA21 BLZF1 ZHX1 SWI5 CAV2 BOLA3 CENPK PPP1R21 MRPL51 NDUFB2 FAM46C DUS1L NEK9 WARS
DMWD POLD1 VIPAS39 BTD SNRPB2 CALD1 MBOAT7 AIG1 ERICH1 CHMP3|RNF103-CHMP3 VWA9 PMS1 NSMAF EIF3E ADSS HAUS7 RAB28 HMGC
KTI12|TXNDC12 GAK SFT2D2 DEK C12orf23 PSPC1 UPF2 ZNF823 CPT1A C6orf226 KIF22 EIF3I NINJ1 EFR3A SUCLG2 FOXF2 SFXN4 MYL6B UBAP2
FBXO6 NCOA7 KCTD3 LIN7C LONRF1 CCP110 ITGAE NXPE3 HMGXB3 C14orf2 WDR83 IFRD2 ANKRD13D COMMD6 KCNMB4 PYCR2 GALNS HIST1H
ACP6|LOC101060704 METTL17 IRF9 MT2A C9orf3 ROR1 LBH SYNM PTDSS2 SLC25A23 TBC1D23 FAM13B HINT1 HEXA SETD4 HNRNPU NRD1 USP
RFC5 MAP7D1 DDX56 MTFR1L AAK1 PEBP1 MRPS18B FBXO2 AOX1 RPL14 CYR61 JRKL C1orf86 LY96 EEA1 ADCK5 IL1R1 KTN1 CTTN MYBBP1A A
SNX15|ARL2-SNX15 GGT5 GULP1 APOC1 PARD6B NEK2 MBD1 RRAGB RCN2 WDR73 STARD7 ARFGAP1 SHMT2 NDUFA5 ZBTB40 AP1S1 NCLN LY
HDLBP PNP SAFB CBWD3|CBWD5|CBWD1|CBWD7|CBWD2|CBWD6 FANCD2 MLLT4 ANKZF1 HMGB1 ACTR2 SENP5 GCSH|LOC729080|LOC641746|G
BAIAP2L1 RCN3 ESPL1 MMS19 C17orf103 B3GNT5 DBNL CHPT1 RPTOR ERGIC1 ARHGEF10L ATOX1 ACTR3 PDCL3P4|PDCL3 UGCG STT3A LPP O
NMB EIF6 MACF1 SEC24D POLR2L MRPL4 MDFIC TRAF3IP2 NUDT4P1 PCMTD2 NUFIP2 ZDHHC2 ADD3 MBNL2 KIAA1279 AXIN1 KANSL2 POLR1C N
RECQL SMARCC2 FAM109A TFDP2 HCP5 CCDC12 ECHDC1 SMARCC1 FOXQ1 C16orf92 C20orf24 FHL3 SPATA5L1 POLG2 SLC37A3 NRM C8orf44-S
C5orf24 IFI27L1 TSPAN3 MFSD6 C8orf58 RHOF SERINC3 EPDR1 PTPLAD1 DERA C11orf75 DTX2|LOC101060822 L3MBTL2 MCRS1 C18orf8 TPP2 GIN
SFPQ DDX19B NGRN EXT2 DIAPH2 CRYZL1 PTPN12 ABL2 LOC727849|LOC388152 MECP2 PSMA1 MAP3K5 RXRA CLDN11 TIMP3 MXRA8 ACACA L
LYPLA1 RPS23 RPP40 UAP1L1 SCOC YOD1 RALY NUP93 SMAD3 YARS2 TBC1D12 APCDD1L HMOX2 ZNF581 CDK12 ANKRD10 PLP2 TAGLN2 CHM
ATP6V0B C5orf28 YTHDF3 RPL7L1 SKP1 LOC100996657|SRSF10 PEX14 THYN1 ZBTB38 PGRMC2 UBE2I OSTF1 FYCO1 FBLIM1 PTCD1|ATP5J2-PTC
SLC25A5 MYO1C RBM23 ARMC1 FAM89B PELO C1orf63 C9orf37 BTN3A2 TMEM66 C17orf59 SLC15A4 PRKCI LEPRE1 OSMR MRPL12 GNPDA1 SOD
TIPARP CHEK1 SCRN2 LRRC58 COPA RTN3 KIF11 SKP2 SYNC ZNF175 PART1 PARVA PSMB6 MAP3K14 PHC3 TRMT112 TNFRSF11B RNF34 GOLPH
HSD17B11 SH3GLB1 HMGN3 NOMO2|NOMO3|LOC101060373|NOMO1 MRPL48 XRCC6 SUPT5H URI1 PAG1 PIK3C3 CAPN14 SOS2 USP16 LARS MR
KNSTRN BCAP31 C12orf52 SLC25A44 PI4K2A PP7080 CXXC1 MED8 TEX261 ZSCAN26 CD40 TCEAL8 GATAD2A PDXK PIH1D1 RAET1G EFTUD2 CC
HSD17B7|HSD17B7P2 COA5 WTIP FOLR1 THAP8 TCF12 ZNF721 SNRPF ARL4C TTC4|HEATR8-TTC4 SCD5 LSG1 PLEKHG2 EMG1 NCEH1 BAG1 AR
LPXN NDUFB4 TP53INP2 PUS1 MYO10 SMYD5 BCL9L RPS13 MASTL RBM19 TAPBP RUNX1 MID1 ATP2C1 CCDC104 TMEM230 AHCY POGK MAPK9
GRSF1 EXOSC9 RPS10|RPS10P7 CTAGE7P DLG3 ZFP36L2 PDRG1 FAM171A1 SSR3 STRADA OSTC|OSTCP1 MAGT1 BATF3 LLGL1 FTL ARIH2 USP
ASPH TMPO KIAA1524 TM7SF2 FLOT2 HDGFRP3 RRAS2 SH3BGRL SRP68 NBPF3|LOC100288142|NBPF15|NBPF8|NBPF12|LOC101060226|LOC1010
PPM1M CDKN2D NRBP1 SLC43A3 STUB1 SLC25A22 MRPS7 MAPK14 PITHD1 CCNL2 GTF2F2 RNF113A RRP36 DPM3 ABTB2 EIF2S3 VBP1 RPSA|RP
KLHDC8B AIM1 MPV17L2 GSN MED11 WWC1 PLEKHA3 ZBTB45 PLEKHG3 CPSF3 FRMD6 TMEM14A CWF19L1 GPKOW YY1AP1 RAB23 SENP6 ARP
ABCC3 SMIM7 TRIM39-RPP21|RPP21 HLA-DMB MRFAP1L1 TMEM209 RRAGA SPR SAMD9L CARS2 RNF5|RNF5P1 ABCE1 ARHGEF3 EXOC8 CSNK1
NR1H2 ZDHHC12 TCTN1 STAT3 C3orf17 PHC2 PPP2R5C SLC38A2 SYNJ2 WDR36 SPHK1 TRIM26 MBD4 CA5BP1 LOC113230 MPZL1 ORAI1 PARPBP
APAF1 TRPT1 SLC36A4 NOP16 TNK2 INPP1 IFNGR2 ANKRD11 SCML1|SCML2 PKP4 NT5DC3 SEZ6L2 DDAH2 STX7 TRIM27 NDUFB5 TMEM214 CDC
GBP2 FBXW9 MTCH1 WDR4 HK1 SLIRP C1orf52 CDC42 C11orf35 VPS33B UACA HEXB LRPPRC CD97 SETD5 PPP1R16A PTER TIMM23B|TIMM23 PC
PRRC2C PCID2 EDF1 LTN1 ARHGEF18 ARRB1 ZCCHC3 BLOC1S3 ITM2C FKBP9|FKBP9L EREG CENPA SUMF2 TMED5 MCAM PARP2 CDCA7 C14or
EIF3F CSE1L LOC101060373|NOMO1|NOMO2|NOMO3 CCNJL TOM1 USP53 PTX3 AATF CENPN CDC20 E2F5 MRPL50 ANG NBPF14|LOC101060684|L
RRP1B PPP1R26 PFKM SGMS1 DKC1 ZNF195 ZNRF2 GART CREBZF THOP1 PRPSAP1 RIOK3 C1orf110 SCARB1 TRIAP1 ARMC10 GCHFR TTC7A G
MT1IP|MT1X|MT1E|MT1A|MT2A|MT1B|MT1L|MT1H NFIC ZNF787 SUN2 PRKAG2 GPAT2 HIAT1 H2AFZ PPT1 SLC35C2 UBE2B MT1X MUC4 NUMBL OM
MTUS1 ARFIP2 KDELR3 SRP19 UROS MFSD1 AKAP17A LRP10 PLEKHF2 BUB1 PIEZO1 FAM173A DYNLRB1 GTF3C2 DIMT1 SPAG4 SOAT1 MAP4K5
ZP3|POMZP3 ZBTB6 TSPAN5 EHBP1 PRR11 OSGIN2 PRNP PDIK1L SKA1 SHMT1 ZRANB2 RPP21|TRIM39-RPP21 TRIP13 RPL36AL SRSF4 HEBP2 G
PTBP1 PTAR1 LPAR2 KIF3B PRICKLE2 SNX7 ZNF335 MCCC2 CPT2 SEC31A HEATR1 IVD FTH1P3|FTH1 SDHA|SDHAP3|SDHAP1 NSDHL PSME1 VPS
ARHGEF40 ISG15 NUP210 CASP3 ITGB3BP ISY1 FAM200B PPAN ZNF429 C1orf233 HDHD1 CCRL2 YWHAB CUL1 LY6G5C PDIA5 DHX9 SUCLG1
NBPF15|NBPF8|NBPF12|LOC101060226|LOC100288142|LOC101060202|LOC100996575|LOC101060238|NBPF11|NBPF24|NBPF16|NBPF3 CNEP1R1 SL
BTBD6 ZNF213 DUSP11 L1CAM SKA3 SH3RF2 TMA16 MST1R TIGD7 PLCXD1 CREB3L2 FOXP4 PPT2 RTN4 MOB3A PGAM1|PGAM4 GFM2 NDUFB10
MRPL18 PGAM5 FBXO21 TUBA4A GRB10 CLSTN1 EEF2 TBL1X PRDX3 CHCHD3 LMAN2 MOV10 RFWD3 INSIG2
NBPF8|LOC101060684|LOC101060362|LOC100996575|NBPF15|NBPF14|NBPF11|LOC100288142|LOC101060202|NBPF24|NBPF16|NBPF12|LOC101060
PILRB IFITM3 SLU7 GPATCH1 VPS37C WDR11 ZNF580 APOBEC3B|APOBEC3A|APOBEC3A_B SAP130 UBE2S PCDH1 FDX1L WDR46 OCIAD2 CDK9
ATP5L NUDT9 EPB41L4A-AS1 STX1A LYN DUSP6 ELP5 MRPL3 INPP4B TOMM6 POMP BMPR2 B3GALNT1 AHSA1 EAPP C17orf58 GCC1 TIMM9 COX
SDCBP PYGL NUCB2 CUL5 SNX27 MTA3 CHTF8 LOC100996579 FPGS NUBPL ORC2 SNRPA1 NUBP1 SFT2D3 MCOLN1 RIN3 AKAP1 RPL19|RPL19P
NPIPL3|LOC101060465|61E3.4|LOC101060576|LOC101060564|LOC100132247|LOC728888|LOC613037 PIGA POLR2K SASS6 PREB HNRNPF BRAT1 S
GTPBP4 LSM5 SURF1 ZBTB1 TUBGCP2 GTF2F1 C16orf13 RPS15A GINS4 TAF1 PPME1 AP1G2 TNPO3 C16orf91 SLC9A6 CKLF CCDC111 PDDC1 TR
SDC1 SERPINB1 MUL1 KIFC2 PTPN21 MOK SLC4A9 TBC1D1 IQGAP1 OTOS FIGNL1 ZNF226 MRPS5 AGPAT2 EMD WIPI1 APH1A CYP27A1 PUS7 CC
HTATIP2 NEMF ZNF841 MED1 CLU FAM86A|FAM86C2P|FAM86C1|FAM86DP|FAM86HP|FAM86EP|FAM86JP DDX6 TFAP2C ENDOD1 DPH2 IDI1 VPS28
COMMD10 GLTSCR1L COPS8 AKAP9 CST6 SACM1L NGLY1 KLHL12 CD63 NCBP2 GPSM3 TOP2A SLC20A1 SIL1 GADD45GIP1 FEN1 CAPRIN2 PGAP
FAM69A IQSEC1 RBM22 BAG2 PLBD1 PSMA7 PAQR5 CHP1|LOC729603 TUBG1 SHROOM3 ELOF1 PLAGL2 HMGCL TRIM38 ACAA1 YWHAE|LOC649
PRSS23 OTUD6A DCP2 MRPL49 RPL18 ISG20 POLR1D SPATA20 ANKRD29 KRR1 PSMB5 MTRNR2L3 C1D KIAA1191 SNHG8 PC USB1 GPN1 MXD3 S
DNTTIP1 CDK5R1 BTN3A3 TMEM116 IKBKE RELA HIF1A MRPL40 QTRTD1 SHFM1 PKMYT1 TSEN34 DUT KIF18A LRRC59 TXNDC9 OTUD5 SLC41A2
FAM43A GSTM3 C19orf54 ARMCX6 EIF3L 6-Mar NFATC2IP GDI2 FAM210A PKN1 SPON2 RHBDF2 SCML1 MLF2 FLJ23867 RAB13 FBXW11 TMEM201
SAP30BP PRMT3 SUPT20H ACBD4 MRS2 CDKN1C DSCR3 ZFAND3 RHBDL2 PPAT HSPH1 SPSB3 ZDHHC14 NIPA1 DPP8 CDC16 MSRA DMAP1 IFI35
C11orf24 CAP2 TIMM44 SLC41A3 PSMD1 TMEM128 MLH3 ILVBL CSNK1G3 IFNGR1 FAM83D NOL9 MBIP IPO11-LRRC70|IPO11 TSPAN6 RNF219 CCD
DEAF1 GLRB HLA-DMA MSLN CIZ1 UBXN6 ZNF700 DDX60L WWTR1 METTL4 CIB1 ZNF680 MBD3 LOC152217 TNFAIP2 SLC35A3 IER3IP1 RPL41 DR
SPANXB1|SPANXB2|SPANXE|SPANXC|SPANXD|SPANXF1|SPANXA2|SPANXA1 PAICS TOMM40L MMD NFKBIE PHF20L1 TCEB2 KCTD7 MCM3AP NIT
NBL1|C1orf151-NBL1 GALNT11 CHAF1B QSER1 FLOT1 NDUFA3 PIGO SLC35E3 LYRM4 CSRP2 HLA-DRB1|HLA-DRB3 CANX HIPK2 GPANK1 DESI2 D
USP22 TMX2 VPS52 ZHX1-C8ORF76|C8orf76 PPM1F GNPAT MYPOP WDR18 E2F6 HSD17B12 TMEM126B DPY19L4 NDUFA9 RSRC2 TBC1D2 FZD6 F
DCAF13|DCAF13P3 SHISA2 ANKRD27 EIF3K CDC42SE2 MALAT1 RRAGD EIF2S2 CKAP5 RNF130 RAB9BP1 CPSF4 ENC1 ZNF28 MAP2K1 PNO1 SUL
KIAA0430 FAM72A|FAM72B|FAM72D|FAM72C|LOC101060656 POP5 ZBTB44 SIX1 TIMP2 PTP4A1 MARS CNPPD1 SREK1 UBR2 PPIE WBP1|INO80B-W
YIPF5 GPRC5B DIS3L STRN4 LCN2 SUMO4 SETX DDR2 WWP2 KCTD12 CBX5 ZNF326 CTSH ASB7 PPP2R5E BOLA1 COL5A1 PAF1 DERL1 TTC9C S
ZNF266 THSD4 VTI1B FEZ2 DLAT TBCD TMEM48 TCEAL3|TCEAL4 TATDN1 PRICKLE1 WNT5B PEG10 KHDRBS1 ZBTB5 ZNF12 SLC25A38 PPP1R18 D
CSRNP2 DPAGT1 PPA1 ST7 MAGED2 MAMDC2 SSBP1 CASP2 ATP5E PRKRIP1|LOC100630923 POPDC3 SERBP1 COA3 TFPI2 GTF2H3 SHKBP1 JUN
TXNDC17 ATP1B3 DDX3X TRIM25 CRKL TYW1B|TYW1 NUP62 AP4E1 RASA3 EPB41L5 CDKN3 PGD ARHGEF10 TMEM168 MPG ZNF133 ITPRIP POL
GUSBP3|LOC101060519|LOC100653061|GUSBP4|GUSBP2|GUSBP1|SMA5|LOC100170939|GUSBP9|GUSB SEPN1 SH2D5 TIGD5 RPL26L1 NUDCD1 P
RPS6KC1 TDP2 DAAM1 SELENBP1 GOLT1B ARHGAP22 TMED4 FOSL1 NPC1 ATG14 ALDH9A1 TTC38 TRAF3IP1 SMARCE1 VRK1 STK17B SUSD1 B
MRPL53 SMEK2 C11orf54 CENPM BTG3 RBL1 PAK2 TCEAL1 CMTM7 FAM64A CENPF SLC25A43 SMAD5 SNORD33 WASF2 CDK10 ABHD16A MDM4 L
ZNF788 RANGAP1 SNCB PUM1 TNNT1 NDUFB9 PDE12 RAB7A SGPP1 ARNT2 CBR1 ITPR3 TRAPPC6B BIRC3 RNLS ZNHIT6 DCAF8 RNGTT CDCA8
ST20|BCL2A1 PYROXD2 POLR2G IFT52 CNBP PDHA1 MMGT1 TKT BYSL HSPB1 INPP5E COX7A2L DDT PMPCA RNF38 ARRB2 U2SURP CMTM8 PAF
FRAS1 F8A1|F8A3|F8A2 MRPL10 ATP13A1 TPX2 ATP9B PRKRA TLN1 MITF MESDC2 CSTF1 TPRG1L PAIP1 BTN2A1 C15orf48 NSMCE4A IGF2BP3 FN
NDUFAF4 RNF14 RAB3IP NDUFAB1 MRPL45 FGFR1 BAK1 COX6C CERK NSMCE2 SPECC1L ZNF208 TTC37 GOT1 RDH14|NT5C1B-RDH14 CASP6 AT
PUF60 ELOVL5 DARS EPS8 SHC1 TANGO2 ARL4A BLOC1S2 SAAL1 HERC2 CACTIN USE1 S1PR2 FAM96B LIMS2 HNMT CAND1 LUC7L2|C7orf55-LU
PALLD SDAD1 RPL35 IMPDH2 ZIC2 MROH1|LOC377711 POM121|POM121C CLTA CCDC106 SMC3 ZNF702P MACROD1 ASB13 GSS LOC101060301|L
PPP3CA FAM63B ZNF181|ZNF302 ZNF529 CES2 ALDH3A2 OXLD1 TMEM191C|TMEM191B|TMEM191A MRPL52 SPTSSA MKNK1 BCLAF1 GOLGA3 C1
KNTC1 EXOSC2 MYL12A RING1 CXorf61 NPC2 LAMTOR2 YTHDF1 EVI2A GTF3C3 HS3ST1 RBPJ LRRC8D METTL2B TRUB2 FGFR1OP2|LOC1003350
PIN1 POLE3 BOP1 RIN2 POLR1E SIPA1 LOC284889 PTK2 BTN3A1 TRIM2 ATAD1 NDUFA8 FABP5 GLTSCR2 SORT1 PLIN2 LLGL2 USP7 YWHAQ OTUD
SLC35D2 SON ALCAM ORAI3 DENND1A FAM76B TMEM99 MZT1 SBNO2 TRAM1 RACGAP1 SLC6A6 TRIM29 UBXN4 NUP37 RHEB RAB35 ERLIN2 C4o
EIF3M MICU1 ZNF24 RAD18 C12orf44 PBDC1 LDHB FUCA2 CCPG1|DYX1C1-CCPG1 BAMBI RAB5B IRF3 PIP4K2B EBPL MRS2P2 MRPS12 PRRC2B C
ERO1L PSMC3IP ABI2 KIAA0317 H3F3B TMEM87B ENDOG EZR TMEM14C CHOC1H1 TMEM33 HINT3 POLDIP3 RPL32 WDR20 CDC6 CST3 TEN1 CSD
CD99 OCRL ARL6IP6 ZCCHC9 GLA HSPA8 DIDO1 LYSMD2 CBFA2T2 MNF1 IMMT NGDN CNOT6 MARK3 SAC3D1 DRAM2 STK40 AGGF1 PRRG1 PIGF
TBC1D9B PNPLA6 RABEP2 VWA1 NOC3L AP3B1 C11orf68 AAMDC TNFSF12 PBX1 PXMP2 SYTL1 LACTB2 ALPP ZNF7 RBM38 DDX24 DGKD
GAA ACSF2 OPA1 MITD1 TRAF2 ECE1 MED18 C6orf62 SH3D19 FAM189B AURKB IFI16 ASAP2 YIPF6 USP4 OSBPL10 LANCL2 NUAK1 ALG6 EFHA1 R
CHAF1A SLC17A9 TPT1 S100A3 MRPS28 FTH1 IER3 KCTD10 TOR1AIP1 CEBPD ALDH6A1 MLL3 NPRL2 GRWD1 BCKDK CTSB CSRP1 MOSPD1 BOD
PDSS1 C1orf198 IKBKG CCDC132 NGFRAP1 NSD1 NEDD4L HAGH IL1A NCAPD3 POLR2M|GCOM1 BRCC3 CNDP2 FYN ERCC1 YPEL5 LRRC42 ITGB
TTC12 SNX13 NOP10 PSMD8 XRCC4 UBALD1 CYB561 CHST10 RABGEF1 MAP1LC3B P2RY8 C17orf89 TRAPPC2 KRT8 AURKAIP1 SLC50A1 LMAN2L
EPB49 CRCP C7orf49 DGAT1 NKIRAS1 CORO1C PQLC2 ATG7 PLIN3 LRR1 MSANTD3 FTSJD1 IGFLR1 MCFD2 IL13RA1 EIF2B2 NHP2L1 SPDL1 ZNF5
C17orf80 GALNT2 COX7B TMEM69 TMEM185B TRAPPC5 ANKRD17 ATXN7 STK3 REPS2 C6orf48 TGIF2 VEZT CCNK CNOT7 ORMDL1 ITGA6 NKAP C
PDCD6IP PSMD2 ZFP90 AP2B1 ATXN2 SLC30A7 CXorf40A|CXorf40B SMCHD1 RRAS NET1 POP7 IPO5 UBE2Q2 LGALS12 GCNT2 ZFPL1 C7orf41 SUR
UBE2V1|TMEM189-UBE2V1 CEBPZ FAM107B IRS2 CLN6 PIM3 KAT8 SRRT SNRPG RBM3 APTX DMTF1 HIST1H2BL|HIST1H2BK|HIST2H2BF|HIST2H2
RPL17 ACOT9 GEMIN4 LRRC45 ADCK2 TSPYL5 LRIG1 FASTKD2 POFUT2 PRKACB PAPD4 SAYSD1 ZNF354A TMC6 NTPCR GXYLT2 RCN1 SF3B2 U
MSANTD3|MSANTD3-TMEFF1 PIK3CD DERL2 UXT TGFB1 PPIAL4D|PPIAL4F|PPIAL4E|PPIAL4G|PPIAL4B|PPIAL4C SMCR7 SLC31A2 USMG5 LRRC8E
ZBED6 C1orf131 ATP6V1E1 TXNDC12 SAR1A FRMD4A ORC5 SPANXD|SPANXE|SPANXA2|SPANXA1|SPANXB1|SPANXC|SPANXB2|SPANXF1 BIRC2 N
PLOD2 CHIT1 RPS24 TTC27 TPST2 UCK1 PYROXD1 MRPL9 RUNDC1 6-Sep RPL24 MAVS YES1 C6orf57 PWWP2A SDHAF1 FAM120AOS NUF2 RCC1
FAM104A COBRA1 ARGLU1 STAT1 C11orf83 HIST2H2AC PTTG1 HYAL2 TMEM62 TBC1D10A RPL13AP20|RPL13A|RPL13AP5 ERGIC3 POLD4 MRFAP1
GHDC WEE1 SSR4 NR2F2 SSFA2 MTERFD1 ZBTB24 NLRX1 FHOD3 BSG SMAP1 TEAD4 AGPAT6 SLC26A6 BBX PDE7B EFEMP1 RERE BIN1 PIGB S

USP40 EEF1A2 MIPEP DUSP23 CD14 ZFX TRIP6 UCHL3 NUP35 CCDC167 WDR45B LRFN4 IFT74 C9orf116 PNISR CNTROB EI24 RAB24 CARHSP1 R
DIAPH3 BUB3 COPB2 CTR9 SLC35E2B|SLC35E2 CCDC107 UBR1 PTTG1IP CDC27 GGH COPS6 MCCC1
LOC401357|LINC00265|LOC100132062|LOC100133161|LOC100132287|LOC101060495|LOC729218|LOC441124|LOC100996903|FLJ45445|LOC100133
PPP1R13L HEBP1 FAM86DP|FAM86HP|FAM86B1|FAM86FP|FAM86C1|FAM86JP|FAM86A|FAM86EP TTC19 GFPT1 MRPS24|URGCP-MRPS24 FAM207A
PPP2R4 PKIG TMED3 PPIL3 IP6K2 RBM34 GALNTL4 RCOR1 MKNK2 U2AF2 FCGRT YWHAG MTFR2 PPIH PRKCZ FAM200A EIF2B4 EIF5B LEO1 CEN
PIGH CMSS1 CDC14B LDLRAP1 HHEX EMC2 GTF2H1 SRSF7 ACOT13 PCNXL4 SSBP3 HECTD1 VKORC1L1 NGEF RPS6 CTNS FAM102A SFXN1 SPF
FAM21A|FAM21B|FAM21C MAPK1 MRM1 CTNND1|TMX2-CTNND1 STAG3L3|STAG3L2|STAG3L1 ATP9A HN1L G3BP2 HNRNPA2B1 RNF216 OARD1 UT
OXSM ZC3HC1 NUPL2 UBE3B PLAT EIF3A COA4 ATG2A PPP2R5B YRDC SPOP PTPRF BLVRA ICMT DANCR SEC24B DAZAP2 SUSD3 BOK MYEOV
DHRS4L2|DHRS4|DHRS4L1 RRP1 DOK1 FSCN1 TMEM136 SH3BP5 TMEM203 CBR3 UBAC2 C19orf52 NECAP1 BNIP1 KIAA0391 IGFBP6 DCLRE1A AR
NPIP|LOC101059938|LOC642778|LOC101060412|LOC100288332|LOC101059953|LOC642799|PKD1P1|LOC101060394|LOC101060253|LOC101060449|L
UFSP2 REXO4 STX18 ZNF142 SLC2A6 MUT CFL1 LOC100653309|PI4KAP2|PI4KA METTL2A PIGQ BRD7 KIAA0146 PSMG1 CEP55 DTYMK ALDH16A1
DYNLL1 CD74 ARFIP1 PLSCR1 RPSA|RPSAP58|RPSAP9|RPSAP52 MGST2 SLC35E1 EVI2B CTPS1 SLC25A11 EHD4 DENND6A EMP3 SYNCRIP LOC
TRA2A ESCO1 RFC1 TUBA1C TRNT1 ASMTL FAM175B CRIPAK TMBIM6 TMEM38B SLC25A52|SLC25A51|LOC494141|SLC25A51P1 AKR1B1 KAL1 NF
LOC442459|RCC2 HLA-DRB1|HLA-DRB3|LOC100507714|LOC100507709|HLA-DRB4 ZCCHC17 NDUFA1 HSPE1 MAPK13 S100A2 C2 NOP14 ASB3|GPR
LIG1 COPS3 PMPCB HYLS1 NBPF15|NBPF14|NBPF11|NBPF8|NBPF12|LOC100288142|LOC101060226|LOC100996575|LOC101060238|NBPF24|NBPF1
C10orf118 WWC3 CDK5RAP3 TCEA1 ARPC4 MPP5 SPA17 LHFP ACY1|ABHD14A-ACY1 C7orf55 CLPX KBTBD4 NPLOC4 ROBO3 RAB34 ARSD PJA1 P
POP1 ERLIN1 JMJD4 RNF24 SLC29A1 HIST1H2BK GLRX ALG8 ST3GAL4 SHOC2 TMEM200B FBXW7 MRPL27 SRC CYB5R3 SETD1B VASN NEDD8-M
PHF15 MAPRE2 RAB11FIP1 ARFGAP3 KIF13A YIF1A PPRC1 DLGAP5 C12orf45 TMEM14B ADIPOR1 WAC-AS1 CLIP4 LUC7L3 RABEPK TFPI PTGES3L
STX10 SEC62 PQLC1 EIF2C2 KRT19 DNTTIP2 ENO1 PCBD1 TIPRL SNORD36A P4HA1 MAN1B1 GOSR2 SP3 AKAP8L LRRC20 UBE2Z RNPS1 COL4A
STS THOC3|LOC728554 DCAF10 KANK2 IL4R SEC11A TMEM123 CCL5 TGIF1 PEX5 SCO2 TP53 LENG1 CCDC69 EPS8L2 MNT PPP6C EMILIN2 TMEM
C7orf73 ZCCHC11 LOX SYPL1 BAG5 RELB BUD13 LAMB2 SDE2 RPS27L MGC2752 TGFBR3 SUCO GTPBP6 CASP9 PPA2 MAP3K7 SRPR UPRT SRA1
RPS6KA1 ZNF746 PTPRM MRPS15 AGAP3 RAB6A|WTH3DI CPOX SPSB1 CNOT8 GORASP2 USP36 ADAM17 RBM4|RBM14-RBM4 LINC00467 TMEM1
TMEM120A FOXO3|FOXO3B S100PBP SLC35F2 BNIP3L ATP10D CFLAR LZIC DCAKD TOR4A KIAA0226 PITRM1 GUK1 FAM210B ERCC5|BIVM-ERCC
IMPDH1 PPIC PRKACA SELM SPRY4 HLA-G|HLA-C ETFB SEPW1 MXD1 NMU ASXL1 TUBB4B IDH1 WDR37 AZI2 RPL8 SNHG1 XRCC5 TBRG4 RAB1A
MRPL42 FBXO38 FASTK LDB1 UBALD2 CD164 ABCC1 LMF2 LOC440563|LOC101060301|LOC649330|HNRNPCL1 IL1B HAX1 TMEM167B NUP205 WD
MMADHC RNF181 ADPGK LARP4 SIAE ZMPSTE24 CWC15 PFDN4 PSMB3 DEDD2 TGFB1I1 MT1A SNRNP200 RPL10L RPS25 TOP2B DNM1L EIF1AD
MED19 LRRC57 SREBF1 RNF11 SCMH1 PAGR1 GPR68 RAB18 UBTF GYG1 POLD3 NFYC|LOC494127 PHB WDYHV1 HSDL2 LPIN1 MKRN1 ANXA7 N
COMMD9 RRP15 GPX1 GALNT1 SBNO1 RSL1D1 SLC39A1 PSME2 ADNP2 DHX58 ARCN1 PLEKHA5 LIMK1 SEC24A POLR2E KIAA0101 IFITM1 B4GAL
ERGIC2 USP28 ASB9 ATP6V0E2 CYBRD1 TRAFD1 MTCH2 MGRN1 CDA ME1 LEPREL1 MRPL30 EPHB2 MIEN1 CALM1 KDSR MCM8 TPI1|TPI1P2 NM
LOC101060198|MALL HNRNPA1 AMIGO2 GPS2 HGS TP53RK EVPL NOL10 EDEM3 RUFY2 IFI27L2 TCOF1 SF3B3 RANBP9 CPNE7 EVL SLC17A5 NUD
PPM1D VPS45 TRMT61A PPP1R7 TP53BP2 SCO1 KAT2B C18orf21 GINS2 PDLIM5 SPHK2 GNPTG BRWD1 GPR108 TRADD EFCAB11 C1orf43 GOLPH
FANCG BCL2L14 CKAP4 IFIT3 MYADM STEAP1 FAM50A PGK1 OAT AGPAT9 CDYL MLXIP PLOD1 MTFR1 NPDC1 RRN3 GPAA1 RABL2B|RABL2A C3o
SUPV3L1 FIBP NBAS YIF1B TBCB DHCR24 MEF2D CNPY2 MYO1B ANKRA2 NBN PANX1 C5orf45 CEP70 WASL ZSCAN18 FAM178A RGMB CYBA TAF
C1R TBC1D14 AGFG1 ERI1 RASSF7 PNKP MRPS35 MATR3 CA13 UGGT1 PACSIN2 SEC23B PRKCDBP DCK RFX5 TXNDC15 SPTLC1 HLTF DHFR|DH
SYNE2 NDUFAF3 SQRDL DOCK2 NUP214 TUG1 NOSIP GABARAP FBXL6 GOLM1 DPYSL2 ARL1 SOX13 IFT46 EXOC5 KCTD14|NDUFC2-KCTD14 CD
KDELC2 DUSP7 CDK11A|CDK11B VSTM2L CEP135 NRP1 SOGA2 ACAD8 APOBEC3A|APOBEC3B|APOBEC3A_B CCDC50 FASN C20orf111 MRPS14 G
AIDA NOM1 GPX7 TGM2 BASP1 STAM ACYP2 ACTL6A LYRM1 CLDN4 SLC22A18 RNF141 GRK6 TBK1 STX2 MANBAL FAM46B NR1H3 VPS26A SLC4
SNRNP70 MTHFR ACAT1 PTOV1 PTPN11 CMIP ZNF274 PHKA2 KDM5C ATF3 C7orf26 UBE2F TSSC1 WDR54 ATP5I MRPS34 NDUFA11 DAP3 RTN4R
GGA3 CAPZA2 ZNF749 LPGAT1 BRMS1 MAPKAP1 THOC2 LGALS1 LOC728392 UBQLN4 TAF1B MFAP3 PCMT1 SRI SOCS6 SLC25A32 MRPL24 WDR
MGST3 BDNF CDKN1B LMBRD1 TEFM BTRC NDUFS8 LSM14A SLC25A16 STEAP3 ACBD6 YEATS2 RPA2 PAK6 ABCA1 CSF1 PQBP1 JTB EDC3 TNF
ATP13A3 COPG1 WDR43 GNG5 ACOX1 FOPNL FAM72A|FAM72B|FAM72C|FAM72D|LOC101060656 CKLF-CMTM1|CKLF SNHG11 KIF5B USP24 POLM
GPI NOP2 ASF1B RAD9A SPOPL TAF2 COPZ1 ADAMTS9 AGPAT3 DHRSX TSEN15 CAMK2D RPS4X DHX33 CCHCR1 RPS27A RAI14 LRFN3 FAM216
BNIP2 DHX30 TBC1D22B PTRH1 HYI GTF3A METRN MRPL54 ARID5B FLNC PRAF2 ARF1 MAU2 KRAS HNRNPH2|RPL36A-HNRNPH2 ERAP1 SPTAN
FNTB|CHURC1-FNTB DCTN3 EVI5 NT5C ZWILCH GET4 LSM10 PSMG2 ZNF639 ZNF407 C11orf58 C12orf10 SUN1 MYL6 RPSA CASD1 LPPR2 SMAGP
RPS17|RPS17L OGG1 HDAC9 ZDHHC24 DYM LITAF TOM1L2 APLP1 ZNF410 ABHD15 METTL15 TMEM165 ZW10 TUBA1B C12orf29 RPS19BP1 C10orf
GOLGA1 PIM1 USP9X IFIT2 MEA1 FAM169A NR3C1 DLX6 C4orf48 NEK7 CORO1B EMP1 TMEM171 PFN2 SERINC2 COG2 TCEA2 TARS YY1 TRAPPC
C17orf75 THTPA SLC2A8 BNIP3 CARD6 MYCBP|GJA9-MYCBP LEPROT CUEDC2 DDX42 TXNRD2 C4orf19 CCDC120 MTHFD2 MIIP SS18L2 RPUSD1 C
LUC7L MARVELD1 ZNF76 GOLGA8B|GOLGA8A RAF1 INF2 SUB1 SGOL1 MRPS33 PRPF6 AKTIP AKIP1 RHNO1 RAMP1 HLA-DPA1 SMIM12 CLDN23 S
COPS2 PRUNE UNC13D RTFDC1 GATAD1 SDC4 CCZ1|CCZ1B FHL2 SUGT1 LOC440354|LOC595101 HIGD1A LTB SNRPD1 NUDT6 NCAPG2 LPCAT2
CBWD3|CBWD5|CBWD1|CBWD7|CBWD2|CBWD6|LOC101060578 CLDND1 ITPKA BTBD2 KRT34|LOC100653049 RDBP GPATCH4 PPP2CB DHRS4|DH
CHUK C1orf122 MYCT1 DENND4B KYNU C11orf95 SRGAP2 GPN3 MPP1 CYBASC3 STARD3NL CD2AP CCDC86 PEX7 RPS26 TTC39C THOC1 TMEM9
FBXO17 PSENEN GFM1 H3F3C BABAM1 FAM168B RAD51C AP5M1 CYB561D2 PARP6 ADAM10 C1orf159 SPEN ZFYVE20 CCSAP LIPA GALC FAM220
DIEXF C10orf35 APOBEC3A|LAIR1 CRIM1 DHTKD1 SH3GLB2 MPHOSPH8 ACO1 FLNB MTIF3 CTH TULP4 MFSD10 FAM45A|FAM45B ZNF302|ZNF181
FAM35DP|FAM35BP|FAM35A SLC12A9 TXNL1 RNF168 PDCD2L CRYBG3 CCNYL1 DDA1 GALNT7 CISD2 MAN2A1 GBA LOC100134091|FRG1B|FRG1|L
PPIA CSNK1D VDAC1 NDOR1 MED6 ERLEC1 CD46 RALGDS ICOSLG PRMT6 ARFGAP2 TAF4 NDUFB6 ADRB2 TYSND1 UTP14A TBP ERI2 NHEJ1 SE
SUMO1|SUMO1P3 TFRC NEDD1 TINF2 TMEM80 OCIAD1 DDX46 9-Sep AK2 VPS13A LHPP TES KAT6A PFDN5
LOC100288142|NBPF15|NBPF8|LOC101060684|LOC101060362|LOC101060226|LOC101060202|LOC100996575|NBPF16|NBPF3|NBPF11|NBPF12|LOC1
ZNF444 PICK1 FBN1 C12orf57 HAUS8 JOSD1 SEMA4B TYRO3 DYNC1I2 FANCF SNX14 LONP2 LDOC1L CCNC TBL2 TADA1 RPS6KB2 ADRM1 PEX6
ANKRD9 CPPED1 ALDOA EXOSC5 FAM92A1 MB21D1 AIMP2 PRRC1 THAP3 IQCB1 LAMA5 TMEM184B HNRNPH3 COA1 ATF4 C3orf37 BMPER EMC7
COASY M6PR LAMC1 DOT1L MAZ CLASP2 SRP9 FAM26F PDCD10 EIF2AK3 COL13A1 SMYD2 CHMP4B TMPRSS2 DCTN6 ACADS PVRL2 ZNF761 C1
KARS ATF7IP CCDC8 FAM131A UFC1 EEF1A1 PSMD14 KDM3B FOXK1 PDCD2 MRPL44 B3GALTL ETF1 MTPAP EIF4G1 UBE4A RPL21|RPL21P28 C1
UBE2G2 NMRAL1 ATP6V0A4 PARP1 ACSS2 COPS7B FGFRL1 BTF3L4 DDX55 GOLGA7 RIN1 FERMT2 SEC23A C2CD5 CLOCK SLC35A4 MLX TMEM8
RPL29|RPL29P2 ZNF45 ACYP1 ARPC2 PHACTR4 AP5Z1 TK1 GAPVD1 TAX1BP1 MTHFS|ST20-MTHFS ZDHHC18 LETM2 SNHG3 C10orf88 HLA-F C9o
ST3GAL2 APIP MOXD1 CCDC84 RPS10-NUDT3|NUDT3 MYLIP PHIP FAM35A HMGN5 ODF2 AARS2 FAM103A1 SLC11A2 SMCR7L DHX32 EPB41 FAM
MED4 IKBIP RMI1 FAM108C1 RNF220 CARM1 TRABD PTPRS SHCBP1 NPHP4 COTL1 MYD88 ITGB1BP1 PML EBAG9 UROD COMMD4 DPP3 DAB2 T
ATAD3A|ATAD3B TLE1 PPARA DVL3 SERTAD3 MGLL SCFD2 ATP5SL MOSPD3 TRAPPC12 NSF TBCC TBC1D13 EBF4 SGK223 PSMG3 LSM6 SMURF
NBPF15|NBPF14|NBPF11|NBPF8|LOC100288142|LOC101060202|NBPF24|NBPF16|LOC101060684|NBPF12|LOC101060362|LOC101060226|LOC100996
TFB2M CPSF1 UGDH S100A4 PXDC1 C2orf29 NUP188 SLC36A1 SDPR LOXL2 CPEB2 ATAD2 LRRK1 NPEPPS DENR RARRES2 DDRGK1 SENP2 CLIC
WIPF2 SMARCD2 TRIM21 SBF2 PPM1B FOXRED2 CYP20A1 GLTSCR1 PEA15 RAB6C|RAB6A ZNF23 NDUFAF2 TBCA GSPT2 TRIM13 FNBP4 ATP6V0
FBXW5 NLRP2 YIPF1 MAP3K2 ANKRD13C TXNDC5|BLOC1S5-TXNDC5 TSPO DLK2 LEPROTL1 CLINT1 SYMPK ZNF451 REEP5 POLE2 FADD ARRDC
PTGR1 ITPA CALU ANKFY1 EMC4 SAP18 ZNF69 CREG1 IFIT1 LIMCH1 SERPINB6 JUNB CMPK1 RWDD1 SSH1 ENSA RHOB SAP30 XPA CKAP2 IGDC
HADH RPAIN TET2 EPG5 TMEM245 SRSF1 RPA1 C5orf44 ACTR1B IFI30 TROVE2 EEF1E1 PLEKHM1 GLUL LAMTOR1 SPIRE1 HK2 MICB ELOVL6 MD
MICAL2 UBA52 PPIF MTRF1L RIPK1 CPNE3 CST1|CST4|CST2 DNPH1 PARP10 GNB2L1 KIAA0195 ICAM1 RBM47 SNORD36C XAGE1D|XAGE1E|XAGE
PNMAL1 CHIC2 ABCF3 SLC16A3 APLP2 ATPAF1 NCBP1 ADK TMEM164 SLC9A1 HDAC2 SRPRB CRYZ TTI2 TGOLN2 GBAS CTBS PIGS MRPS27 EDC
TMEM234 VAMP3 CD81 CHORDC1|LOC727896 TIMM8B TCIRG1 SH2B3 TRIM44 STAP2 TMCO1 TRIM39 PAPSS1 PARL C19orf33 SIDT2 TMEM231 GR
DDX54 TRIP4 SNX3 FGFR1OP PTPRA STOML2 HSF1 ADSL FTSJ1 DR1 BAP1 GLRX5 FLAD1 CAB39 RAB5C SNORA25 TMEM161A PIP4K2C CYLD ME
CAPN5 USP48 RRM1 PA2G4 C1orf35 NEDD8 MFSD12 CTDSPL RUSC2 MRPS2 POLR3H ABHD11 NSRP1 RAD17 KLHL29 RILP CKLF|CKLF-CMTM1 TS
CMBL XKR8 SRSF2 CNKSR1 CEP41 SDHC GNB5 PRKAR1B AAED1 GBF1 UBTD2 VCL TGIF2-C20orf24|C20orf24 FYTTD1 TCF3 HFE SERPINE1 EIF2A
SSSCA1 PRPF38A STAT2 EDARADD DTL PORCN ALG3 TMEM194A CENPV RNF40 CREBRF PDCD11 CTBP2 TM7SF3 LLPH LRRFIP2 CYTH3 INTS4 M
SLC45A3 GXYLT1 C6orf132 C8orf76 OXSR1 TMEM219 BCCIP RAD51 RPL13A RAD51D ACN9 DLD KIAA2013 SARDH PPFIA1 RANGRF HMMR TMEM3
SLC39A11 LY6E GCAT GSTO1 SRD5A1|SRD5A1P1 HNRNPA3 MAD2L1BP ENPP1 LAPTM5 CETN2 PITPNM3 NOLC1 SCNN1A NRBP2 IARS2 IDUA RHC
PDCL3|PDCL3P4 UBIAD1 CYB561D1 FAM217B TMEM55A NPTN TMEM258 RNMTL1 SCAMP2 PLD3 RTKN SART3 SGSM2 UPP1 FOXP1 APOA1BP ND
FAM114A1 TJP2 KRT10 SLC9A8 RAB27A CDCP1 ARF3 CSF2RA PEX19 TXNRD3 CNP YAP1 NNT PSMD7 SC5DL SSX2IP ITPKC SIK3 RAD21 SNRPB R
SPANXD|SPANXB1|SPANXB2|SPANXE|SPANXC|SPANXF1|SPANXA2|SPANXA1 C4BPB ORC6 PFN1 ZNF654 POMT2 SS18L1 SGOL2 ACD BCL3 MKI67I
MORN2 P4HB TTPAL RBM27 HIATL1 ZNF282 FAM160B2 CERS2 WDR55 NUMA1 CERCAM TMEM254 C11orf31 SUPT6H GIPC1 ELL2 ZNF330 PVRL3 T
ANKRD1 SAA2 ATP6V1D DDR1 PMEPA1 ADPRHL2 FBXO18 ZZZ3 TMEM185A IGFBP4 TMED10|TMED10P1 SNRNP40 UBE3C HLA-A|HLA-H PDSS2 ND
DCTN5 TEX30 B3GALNT2 HKDC1 CLNS1A PSME3 NUP43 ZXDB FUNDC2 NME3 EPS15L1 TNFRSF6B|RTEL1-TNFRSF6B MGEA5 UBR7 NMD3 BCAT1
ANXA4 ALKBH3 ZFYVE19 UAP1 PARD3 IRAK4 ECI1 KDM3A RPS14 MLPH ATIC APBB1IP CXCL16 RETSAT ADAM15 NRG1 CAST TBC1D9 TTK TGFBI
SPINT2 PIAS3|LOC101060431 CASK GIT2 NCAPG PXK ATP6V0E1 DKFZP586I1420 TIFA UTP11L CD274 BAX FDPS LRPAP1 SLC52A2 G6PC3 RPL37 S
FUNDC1 AMD1 GGA2 PRKCA TRIB2 CCDC115 PRMT7 SDCCAG3 TTC1 COQ2 SRF KHDC1 ATP8B1 HIPK1 MAT2A MAL2 C2orf74 CLN3 STX4 SEMA4C
VDAC2 VHL IFI44 RPS2P32|RPS2 ZNF358 TMA7 RPS6KA3 NUSAP1 LANCL1 FAM49B C15orf40 ATP5G1 PPWD1 ERCC2 TRMU BET1 CCDC9 USP3 VP
RNF169 CTSC MPST

Disclaimer
We made every attempt to ensure the accuracy and reliability of the results provided through this webservice. However, the information is provided "as is" without responsibility
image(s) using our serivce.
