# Supplementary material for: Tribbles Homolog 3 Involved in Radiation Response of Triple Negative Breast Cancer Cells by Regulating Notch1 Activation
Source: Cancers (Basel). 2019 Jan 22;11(2):127. doi: 10.3390/cancers11020127 (PMC6406679; doi:10.3390/cancers11020127)
Supplement: Supplementary file 1 [file cancers-11-00127-s001.zip › Supplementary data_proof_20190122.pdf]

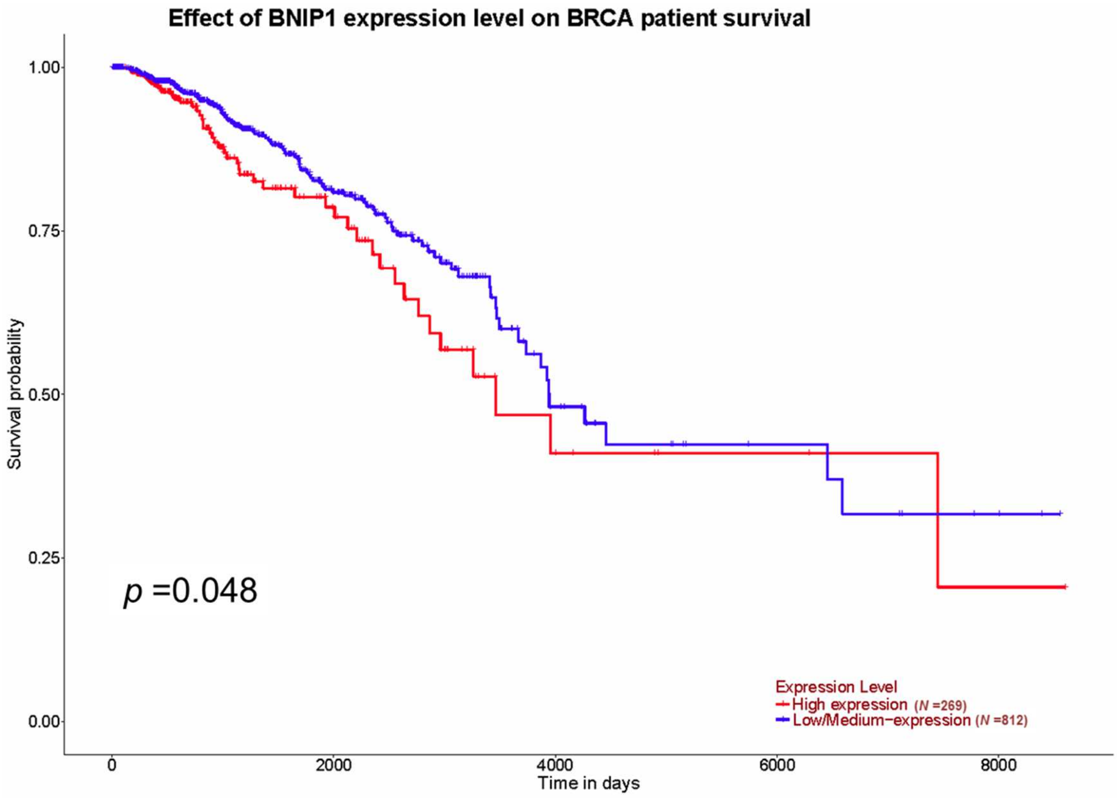

Figure S1. The correlation of BNIP1 mRNA expression on breast cancer patients overall survival. Effect of BNIP1 mRNA expression level on breast cancer (BRCA) patients overall survival was obtained from UALCAN web based analysis tool by analyzing TCGA's data.

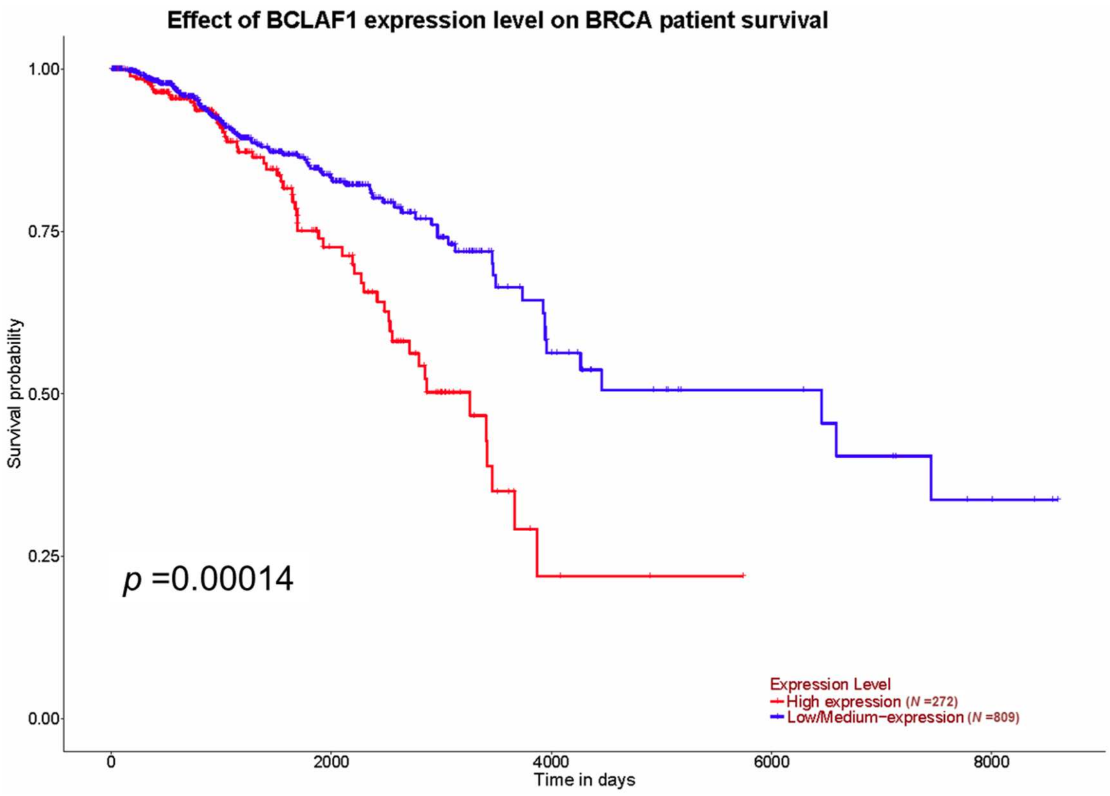

Figure S2: The correlation of BCLAF1 mRNA expression on breast cancer patients overall survival. Effect of BCLAF1 mRNA expression level on breast cancer (BRCA) patients overall survival was obtained from UALCAN web based analysis tool by analyzing TCGA's data.
